# Transduction of group I mGluR-mediated synaptic plasticity by β-arrestin2 signalling

**DOI:** 10.1038/ncomms13571

**Published:** 2016-11-25

**Authors:** Andrew G. Eng, Daniel A. Kelver, Tristan P. Hedrick, Geoffrey T. Swanson

**Affiliations:** 1 Department of Pharmacology, Northwestern University Feinberg School of Medicine, Chicago, Illinois 60611, USA

## Abstract

Conventional signalling by the group I metabotropic glutamate receptors, mGluR1 and mGluR5, occurs through G-protein coupling, but evidence suggests they might also utilize other, non-canonical effector pathways. Here we test whether group I mGluRs require β-arrestin signalling during specific forms of plasticity at hippocampal excitatory synapses. We find that genetic ablation of β-arrestin2, but not β-arrestin1, results in deficits in plasticity mediated by mGlu1 receptors in CA3 pyramidal neurons and by mGlu5 receptors in CA1 pyramidal neurons. Pharmacological studies additionally support roles for Src kinases and MAPK/ERK downstream of β-arrestin2 in CA3 neurons. mGluR1 modulation of intrinsic conductances is otherwise preserved in β-arrestin2^−/−^ mice with the exception of a rebound depolarization, and non-mGluR-mediated long-term potentiation is unaltered. These results reveal a signalling pathway engaged by group I mGluRs to effect changes in synaptic and cell intrinsic physiology dependent upon β-arrestin rather than G proteins. Pharmacological manipulation of mGluRs with effector-biased ligands could lead to novel therapies to treat neurological disease.

## Abstract (web summary)

mGluRs are known to undergo non-canonical signalling regulation, although the underlying mechanisms are unclear. Here, the authors identify a role for β-arrestin2, but not β-arrestin1, in group I mGluR-mediated plasticity at hippocampal synapses.

## Graphical abstract

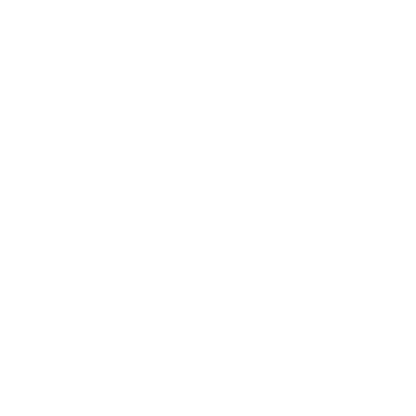

Group I metabotropic glutamate receptors (mGluR1 and mGluR5) function as modulators of neuronal physiology and synaptic transmission and have been the target of therapeutic drug development for pathologies including Fragile X syndrome, epilepsy and pain^1^. mGlu1/5 receptors are seven transmembrane receptors (7TMRs) that differ from other mGluRs in their preferential coupling to Gα_q/11_ second messenger pathways, yet mGlu1 receptors in particular exhibit an intriguing ability to drive cellular responses even when G-protein activity is inhibited pharmacologically^2^,^3^,^4^,^5^,^6^. G-protein-independent signalling by other 7TM receptors (for example, angiotensin type 1A receptors and β_2_-adrenergic receptors) is transduced by kinase cascades whose activity is initiated by 7TM receptor coupling to the cytoplasmic scaffolding proteins β-arrestin1 and 2^7^,^8^, but how the arrestins participate in mGluR signalling and specifically in modification of neuronal synaptic and intrinsic activity remains unclear.

mGlu1 and mGlu5 receptors are widely distributed among excitatory synapses in the central nervous system and respond to glutamate most notably by G protein-mediated stimulation of phosphoinositide hydrolysis, mobilization of calcium from intracellular stores, and activation of protein kinase C. Protein kinase C has a well-established role in some forms of synaptic plasticity that include long-term depression (LTD) of cerebellar parallel fibre-Purkinje cell synaptic transmission^9^ and mGluR5-mediated selective long-term potentiation (LTP) of *N*-methyl-D-aspartate (NMDA) receptor-mediated currents at hippocampal mossy fibre-CA3 synapses^10^. G protein-dependent signalling by mGluR1/5 also alters neuronal excitability, in some instances through slow currents carried by transient receptor potential (TRP)-like channels^11^. Not all mGluR1/5 signalling appears to be mediated by G proteins, however. Some aspects of mGluR1 signalling are intact in conditions where G proteins were pharmacologically inhibited using non-hydrolyzable forms of GDP or *N*-ethylmaleimide^4^,^5^,^6^. GDPβS-resistant signalling by mGlu1 receptors in CA3 pyramidal neurons, for example, underlies transient potentiation of NMDA currents and intermediate-term potentiation of transmission at mossy fibre synapses^2^,^3^.

In this study, we asked whether β-arrestin scaffolding proteins serve as molecular substrates of mGluR synaptic and intrinsic plasticity in a G protein-independent manner. We found that at least two distinct forms of group I mGluR-dependent hippocampal synaptic plasticity were abrogated in β-arrestin2^−/−^ but not β-arrestin1^−/−^ mice. In pharmacological antagonism studies, we identify mitogen-activated protein kinase (MAPK)/extracellular signal-regulated kinase (ERK) and Src as downstream kinases in an mGluR1-β-arrestin2 signalling network. Non-synaptic functions of mGlu1 receptors were differentially impacted in β-arrestin2^−/−^ mice. Our data demonstrate that group I mGluRs employ a β-arrestin2-mediated signalling mode to alter synaptic strength and suggest that selective targeting of mGluR signalling modalities could be a viable therapeutic strategy in the future.

## Results

### mGluR1 plasticity at CA3 synapses is β-arrestin2 dependent

**Figure 1:**
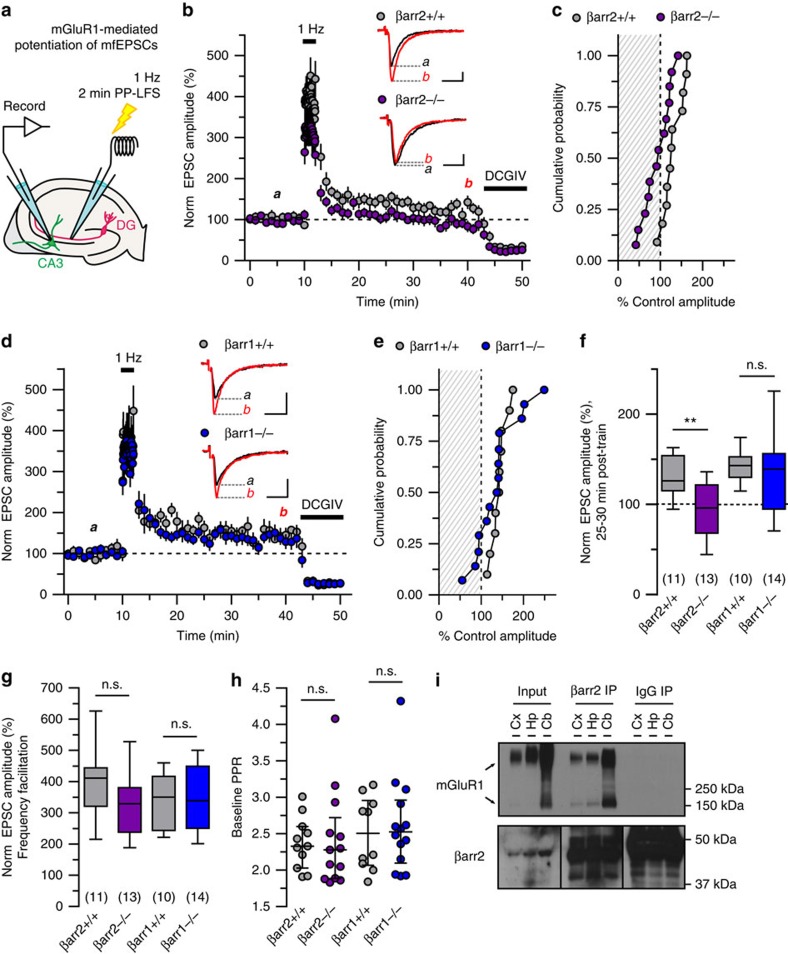
β-arrestin2 is required for PP-LFS potentiation of mfEPSCs. (**a**) Whole-cell voltage clamp recordings in acute hippocampal slices. Paired pulse low-frequency stimulation (PP-LFS) of mossy fibres for 2 min elicits mGluR1-dependent potentiation of mfEPSCs^3^. (**b**,**c**) PP-LFS potentiates mfEPSC amplitudes in βarr2^+/+^ but not βarr2^−/−^ mice. Mean±s.e.m. of pooled, normalized mfEPSCs evoked at basal frequency (0.05 Hz) and 1 Hz train frequency are plotted against time. For illustrative purposes, every third mfEPSC is shown. In βarr2^+/+^ animals (*n*=11 cells, 10 animals), mfEPSC amplitudes measured 25–30 min post-train were elevated compared with βarr2^−/−^post-train mfEPSCs (*n*=13 cells, 12 animals) (*P*=0.009). DCG-IV (1 μM) inhibited mfEPSCs in both the groups. A cumulative probability histogram shows the post-PP-LFS amplitudes measured from individual experiments. (**d**,**e**) mfEPSCs in βarr1^+/+^ (*n*=10 cells, seven animals) and βarr1^−/−^ (*n*=14 cells, 11 mice) mice potentiate normally during and following PP-LFS (*P*=0.22), illustrated by a time course plot of population data and a cumulative probability histogram. (**f**) A box and 10–90% whisker plot of pooled post-train values illustrate normal potentiation of mfEPSCs in βarr1^−/−^, but abolished plasticity in βarr2^−/−^ slices compared with wild type. (**g**,**h**) Frequency facilitation of βarr2^+/+^ and βarr2^−/−^ as well as βarr 1^+/+^ and βarr1^−/−^ mfEPSCs were not different (*P*=0.13, *P*=0.93, respectively); baseline PPRs were also not different (*P*=0.62, *P*=0.91, respectively). Median and interquartile ranges are provided. (**i**) mGluR1 co-immunoprecipitates with βarr2 in wild-type cortex (Cx), hippocampus (Hp) and cerebellum (Cb), but not with an IgG isotype control antibody (*n*=3 replicated observations). Positive βarr2 immunoreactivity was observed in immunoprecipitation and lysate preparations loaded into the same gel. Blotting for βarr2 in the rabbit anti-IgG antibody resulted in strong signal ∼50–51 kDa during short exposures, consistent with detection of the rabbit IgG heavy chain by the goat anti-rabbit secondary antibody. Representative baseline (black, ‘**a**') and post-train (red, ‘**b**') traces are calibrated as: *x* axes, 10 ms; *y* axes, 250 pA. Groups were compared by Mann–Whitney tests. Asterisks denote significance (*P<0.05, **P<0.01); n.s., non-significant.

We initially tested the contribution of β-arrestins to a selectively mGluR1-dependent form of intermediate-term potentiation of excitatory post-synaptic currents at mossy fibre-CA3 synapses. mGluR1 dependence was previously characterized using mGluR1^−/−^ mice and selective antagonists of mGlu1 and mGlu5 receptors^3^. In the current study, we compared electrophysiological recordings from acute hippocampal slices prepared from wild-type and β-arrestin (βarr) knockout mice. Baseline mossy fibre-excitatory post-synaptic current(mfEPSC) amplitudes were obtained by delivering pairs of electrical stimuli to mossy fibre axons in the stratum lucidum at a basal frequency (0.05 Hz) for 10 min. mGluR1-dependent potentiation of excitatory post-synaptic currents (EPSCs) was induced by delivering paired pulses at 1 Hz for 2 min (paired pulse low-frequency stimulation, PP-LFS; Fig. 1a). mfEPSCs were then evoked at basal frequency for 30 min after the train. At the conclusion of each mossy fibre-CA3 plasticity experiment, we verified contribution from mossy fibre inputs by selectively inhibiting mossy fibre transmission with group II mGluR agonist (2*S*,2′*R*,3′*R*)-2-(2′,3′-dicarboxycyclopropyl) glycine (DCG-IV, 1 μM). All data collection, *post-hoc* analysis, and imposition of inclusion criteria were performed with the experimenter blinded to genotype.

Consistent with previous observations, EPSC amplitudes measured in wild-type animals 25–30 min post-train remained elevated when compared with baseline values (131±7% of baseline amplitudes in βarr2^+/+^ animals, *n*=11) (ref. 3). However, mGluR1-mediated prolonged potentiation was absent in βarr2^−/−^ animals, such that post-train amplitudes were reduced compared with wild type (94±9% of baseline amplitudes, *n*=13, *P*=0.009, Fig. 1b,f). Normalized post-train amplitudes measured in individual experiments were plotted in a cumulative probability histogram (Fig. 1c). Neither frequency facilitation during the train (400±34% for βarr2^+/+^, *n*=11, 325±26% for βarr2^−/−^, *n*=13; *P*=0.13) nor baseline paired pulse ratios (PPRs) were different in recordings from βarr2^−/−^ and βarr2^+/+^ littermates (2.4±0.1 in βarr2^+/+^, *n*=11; 2.4±0.2 in βarr2^−/−^ animals, *n*=13; *P*=0.54, Fig. 1g).

In contrast, PP-LFS-induced potentiation of mfEPSCs was intact in βarr1^−/−^ mice (143±6% for βarr1^+/+^, *n*=10; 137±14% for βarr 1^−/−^; *n*=14, *P*=0.22, Fig. 1d–f). Pre-train PPRs were not different between βarr1^+/+^ and βarr1^−/−^ groups (2.5±0.2 in βarr1^+/+^, *n*=10; 2.6±0.2 in βarr1^−/−^ animals, *n*=14; *P*=0.91, Fig. 1g), as was frequency facilitation at 1 Hz (336±23% for βarr1^+/+^, *n*=10, 345±29% for βarr1^−/−^, *n*=14; *P*=0.93). These data demonstrate that PP-LFS, mGluR1-mediated plasticity of mossy fibre-CA3 synapses is dependent specifically on βarr2.

### mGlu1 receptors and β-arrestin2 associate in brain tissue

We tested whether βarr2 and mGlu1 receptors associate in native tissue in immunoprecipitation experiments using brain tissue homogenates prepared from wild-type and mGluR1^−/−^ mice. βarr2, a 46 kDa protein, readily co-immunoprecipitated mGlu1 receptor protein, which resolved as monomers (142 kDa) and dimers (>250 kDa), in the cortex, hippocampus and cerebellum of wild-type mice. In control experiments, mGluR1 immunoreactivity was absent when an IgG isotype antibody was used for immunoprecipitation (*n*=3, representative blot in Fig. 1h, uncropped blot in Supplementary Fig. 1) or when βarr2 immunoprecipitation was performed on brain membrane preparations from mGluR1^−/−^ mice (*n*=3, representative blot in Supplementary Fig. 2). Immunoblotting of lysates confirmed the presence of mGlu1 receptors in wild type but not mGluR1^−/−^ tissue, whereas βarr2 was detected in immunoprecipitation and input conditions for both genotypes. These results support an association between βarr2 and mGlu1 receptors in neurons.

### Role of Src and other kinases in mGluR1-mediated plasticity

**Figure 2:**
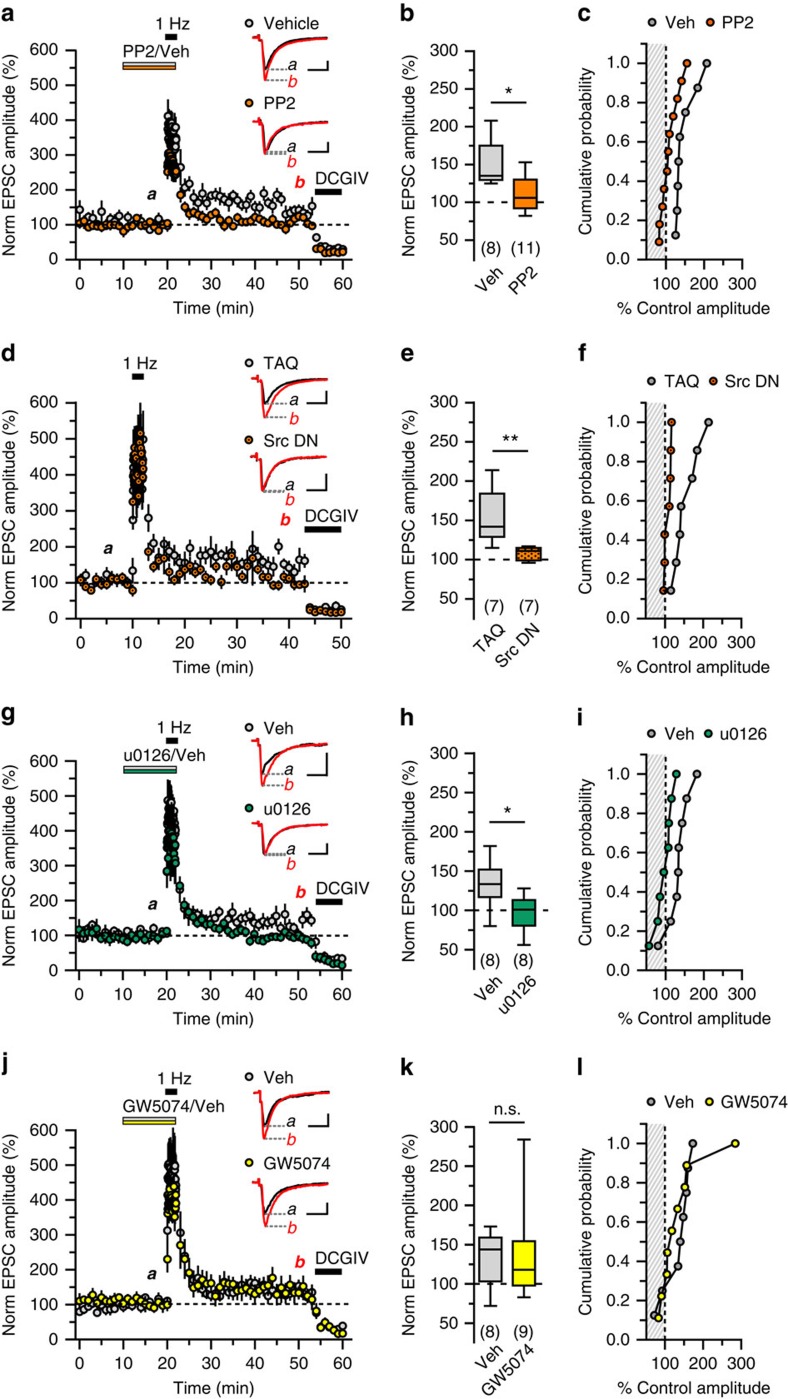
Kinases involved in potentiation of mfEPSCs. (**a**–**c**) Src-family tyrosine kinase antagonist PP2 attenuates potentiation of mfEPSCs by PP-LFS. Either 0.2% dimethylsulphoxide (Vehicle/Veh) or PP2 (20 μM) was applied before and during the train (*P*=0.012, *n*=11 PP2-treated cells and eight vehicle cells from 14 animals). A cumulative probability histogram and 10–90% box plot summarize the population data for post-train mfEPSC amplitudes. (**d**–**f**) pp60^c-Src^ 521–533, a peptide inhibitor of Src, also attenuates potentiation of mfEPSCs by PP-LFS when provided in the recording microelectrode (*P*=0.004, *n*=7 cells using TAQ internal from six animals, *n*=7 cells from seven animals with pp60^c-Src^). (**g**–**i**) MEK1/2 antagonist u0126 (20 μM) prevents potentiation of mfEPSCs by PP-LFS (*P*=0.010, *n*=8 cells treated with u0126 and *n*=8 vehicle-treated cells from 13 total animals). (**j**–**l**) b-Raf 1 and c-Raf 1 antagonist GW5074 (1 μM) has no effect on plasticity induced by PP-LFS, compared with a vehicle control (*P*=0.54, *n*=9 cells treated with GW5074, *n*=8 vehicle-treated cells from 15 total animals). Calibration of representative traces: *x* axes, 10 ms; *y* axes, 250 pA. Groups were compared by Mann–Whitney tests. Asterisks denote significant differences between treatment groups (*P<0.05, **P<0.01); n.s., non-significant.

PP-LFS of mossy fibre-CA3 synapses induces plasticity that selectively requires mGlu1 receptors and βarr2, and consequently we sought to test the role of downstream constituents associated with βarr2 signalling. Src family kinases seemed likely candidates given their association with β-arrestins following β_2_-adrenergic receptor activation^7^, functional linkage between Src and mGlu1 receptors^2^,^6^, and genistein sensitivity of PP-LFS-induced plasticity of mfEPSCs^3^. Bath application of PP2 (20 μM), a potent and selective antagonist of Src family tyrosine kinases^12^, for 10 min before and during PP-LFS attenuated potentiation of mfEPSC amplitudes 25–30 min after the train (111±7% relative to amplitudes measured 5–10 min after drug wash-in, *n*=11), whereas mfEPSCs recorded in vehicle (0.2% dimethylsulphoxide)-treated slices remained elevated (150±11% relative to vehicle baseline, *n*=8; *P*=0.012, Fig. 2a–c). To resolve the pre- or post-synaptic localization of Src activated by the PP-LFS stimulus, the experiments were repeated with a peptide inhibitor of c-Src (pp60^cSrc^ 521–533, phosphorylated at F527, 200 μM) in the internal pipette solution. PP-LFS stimulation failed to potentiate mfEPSCs in the presence of the Src inhibitor (107±3%, *n*=7) compared with the control internal recording solution (156±13%, *n*=7; *P*=0.004, Fig. 2d–f), isolating the actions of Src to the post-synaptic CA3 neuron.

The ERK module of the MAPK cascade has also been implicated downstream of group I mGlu receptors, and evidence suggests that mGluR1-βarr1 signalling promotes ERK phosphorylation in cell lines^13^,^14^. We tested the role of ERK and its upstream kinase MEK using bath application of the selective, non-competitive MEK1/2 antagonist u0126 (20 μM). Application of u0126 10 min before and throughout PP-LFS prevented induction of mGluR1-mediated plasticity of mf-CA3 synapses; antagonist-treated slices exhibited post-train mfEPSC amplitudes that were 97±8% of drug baseline (*n*=8; compared with 134±11% of baseline for vehicle, *n*=8; *P*=0.010, Fig. 2g–i).

c-Raf kinase facilitates activation of MAPK/ERK via association with βarr2 in cell lines^15^ and, therefore, might have a role in synaptic plasticity mediated by mGlu1 receptors. We tested this hypothesis by bath applying GW5074 (1 μM), a potent inhibitor of c-Raf and b-Raf kinases, 10 min before and during PP-LFS^12^. This compound did not affect potentiation (mfEPSC amplitudes 137±20% relative to drug baseline, *n*=9, compared with 135±12% of baseline for vehicle, *n*=18, *P*=0.54) (Fig. 2j–l). This was a surprising result given that the preceding u0126 data clearly support a role for MEK. While canonical models of MAPK signalling cascade proceed through sequential activation of Ras, Raf, MEK and ERK, MEK activation through Raf-independent mechanisms, such as mixed-lineage kinases, phosphoinositide-dependent protein kinase-1 and protein kinase A, has described in non-neuronal cells^16^,^17^,^18^. Our data are also consistent with unconventional activation of MEK as a critical signalling component for induction of PP-LFS-induced plasticity of mossy fibre-CA3 synapses.

### LTP of mossy fibre-CA3 synapses does not require β-arrestins

**Figure 3:**
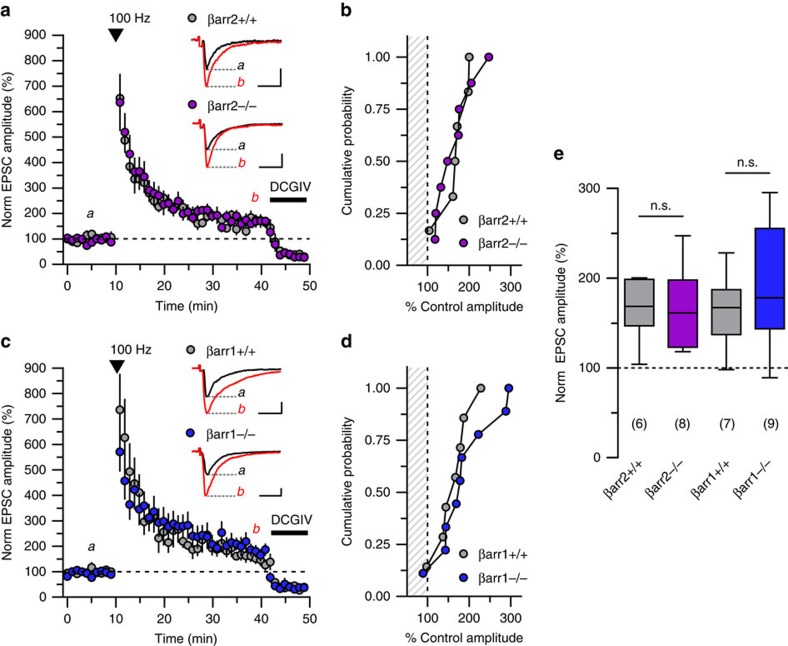
Classical mossy fibre LTP does not require β-arrestins. (**a**,**b**) High-frequency stimulation (HFS) of mossy fibre inputs elicits robust potentiation of mfEPSCs in slice recordings from both βarr2^−/−^ and βarr2^+/+^ mice (*P*=0.85, *n*=6 cells from three wild-type animals, *n*=8 cells from five knockout animals). A time course plot and cumulative probability histogram illustrate robust facilitation following HFS (amplitudes during HFS were omitted). Amplitudes deprecate over time but remain elevated relative to baseline 25–30 min post-tetanus. (**c**,**d**) mfLTP is intact in βarr1^−/−^ mice. mfEPSC amplitudes measured from βarr1^+/+^ and βarr1^−/−^ mice are strongly potentiated immediately following tetanus and slowly deprecate to similar levels above baseline 30 min later (*P*=0.56, *n*=7 cells from six wild-type animals, *n*=9 cells from eight knockout animals). Individual post-train measurements are provided in a cumulative probability histogram. (**e**) mfLTP potentiation levels in β-arrestin knockout mice and wild-type littermates are summarized by a box plot and 10–90th percentile whiskers. Representative trace calibration: *x* axes, 10 ms; *y* axes, 250 pA. Groups were compared by Mann–Whitney tests; n.s., non-significant.

Classical long-term potentiation of hippocampal mossy fibre-CA3 synapses (mfLTP) is not dependent on group I mGluRs^19^, therefore, we predicted this form of plasticity would be normal in βarr2^−/−^ mice. We tested this prediction by evoking mfLTP with three 1 s trains of 100 Hz stimulation, a standard high-frequency stimulation (HFS) paradigm, in CA3 neurons from βarr1^−/−^, βarr2^−/−^, and wild-type littermate control mice. The mean levels of potentiation measured 25–30 min post-tetanus were equivalent between βarr2^+/+^ and βarr2^−/−^ animals (167±14% relative to baseline for βarr2^+/+^, *n*=6; 165±36% relative to baseline for βarr2^−/−^ mice, *n*=8; *P*=1.0, Fig. 3a,b,e). Potentiation in βarr1^−/−^ mice also was equivalent to wild-type littermates (163±16% relative to baseline for βarr1^+/+^, *n*=7; 190±23% relative to baseline for βarr1^−/−^ animals, *n*=9, *P*=0.56, Fig. 3c–e). Pre-train PPRs measured in βarr1 and βarr2 knockout recordings again were indistinguishable from those obtained from wild-type littermates (2.2±0.1 for βarr2^+/+^, *n*=6, 2.3±0.2 for βarr2^−/−^, *n*=8, *P*=0.65; 2.4±0.2 for βarr1^+/+^, *n*=7, 2.3±0.1 for βarr1^−/−^, *n*=9, *P*=0.63). These data demonstrate that robust potentiation of mfEPSCs by HFS is unaffected by the absence of βarr1 or βarr2.

### Non-synaptic mGlu1 activity and β-arrestin2 signalling

We next tested the contribution of β-arrestin2 to mGluR1-dependent modulation of spiking characteristics in CA3 pyramidal neurons. Pharmacological activation of mGlu1 receptors persistently suppresses fast after-depolarizing potentials (ADP) through an unknown mechanism that is insensitive to Kv7 channel inhibition and buffering of intracellular calcium^20^. Postspiking conductances are important determinants of rate and pattern of neuronal burst firing, such that mGluR1-mediated suppression of the ADP effectively reduces the initial frequency of action potential firing during a prolonged suprathreshold current injection. Activation of mGluRs in CA3 neurons can elicit a biphasic, outward current followed by an inward current^21^, and the inward, depolarizing current is supported by a G protein-dependent mechanism^11^. Through these studies we sought to examine a potential role for β-arrestin2 in non-synaptic processes mediated by mGlu1 receptors, and to concurrently determine whether mGlu1 receptors are present and functional on the plasma membrane of CA3 pyramidal neurons in βarr2^−/−^ mice.

**Figure 4:**
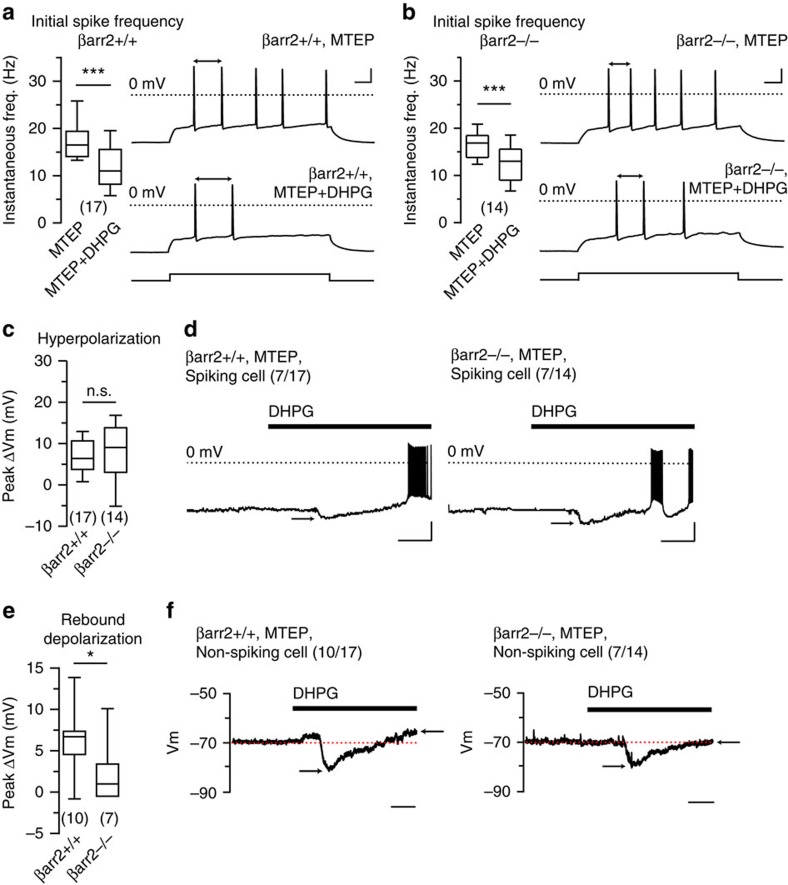
Cell-intrinsic activities differentially require β-arrestin2. (**a**) After DHPG (50 μM) application, the instantaneous frequency of the first two action potentials elicited during a 500 ms suprathreshold current step is reduced in βarr2^+/+^ recordings (*P*=0.0003, *n*=17 cells from nine mice). Action potentials were evoked in CA3 neurons in the presence of mGluR5 antagonist MTEP (1 μM) and AMPA/kainate receptor antagonist CNQX (50 μM). Instantaneous frequency measured during vehicle (MTEP) and DHPG application (MTEP+DHPG) are summarized in a box plot. Representative βarr2^+/+^ traces before and after DHPG application are shown with double-headed arrows denoting the intervals used to calculate instantaneous frequency. (**b**) In βarr2^−/−^ recordings, DHPG activation of mGlu1 receptors reduces the instantaneous frequency of action potential firing during a 500 ms current step (*P*=0.0023, *n*=14 cells from seven animals). Vehicle (MTEP) and DHPG (MTEP+DHPG) instantaneous frequencies are summarized in a box plot. Representative traces from a βarr2^−/−^ CA3 pyramidal neuron show action potentials evoked by suprathreshold current injection under vehicle and DHPG conditions. (**c**) Hyperpolarization among spiking and non-spiking cells elicited during DHPG application is normal in βarr2^−/−^ mice (*P*=0.62), as shown in a box plot. (**d**) βarr2^+/+^ and βarr2^−/−^ CA3 neurons exposed to DHPG (black bar) exhibited a brief hyperpolarizing potential. Representative traces of spiking cells are provided, and black arrows denote the peak hyperpolarization. (**e**) A subset of βarr2^+/+^ CA3 neurons exhibit a rebound depolarization with prolonged application of DHPG that did not lead to spiking, but in βarr2^−/−^ CA3 non-spiking neurons, the rebound depolarization is reduced or absent (*P*=0.047). Rebound depolarization measured in only the non-spiking population of cells is shown in a box plot. (**f**) Representative traces from non-spiking βarr2^+/+^ and βarr2^−/−^ CA3 neurons are provided. Black arrows denote peak hyperpolarization and subsequent rebound depolarization. Current step trace calibration: *x* axes, 50 ms; *y* axes, 20 mV, 600 pA. Membrane voltage traces are calibrated as: *x* axes, 1 min; *y* axes, 25 mV. Instantaneous frequencies altered by DHPG within genotypes were compared by Wilcoxon tests; hyperpolarization and rebound depolarizations compared between genotypes were analysed by Mann–Whitney tests. Asterisks denote significance (*P<0.05, ***P<0.001); n.s., non-significant.

The impact of βarr2 on spike frequency was tested by eliciting a train of action potentials with a 500 ms somatic current injection before and after (*S*)-3,5-dihydroxyphenylglycine (DHPG) application (50 μM, 5 min) in the presence of mGluR5 antagonist 3-((2-methyl-4-thiazolyl)ethynyl)pyridine (MTEP, 1 μM) and α-amino-3-hydroxy-5-methyl-4-isoxazolepropionic acid (AMPA)/kainate receptor antagonist 6-cyano-7-nitroquinoxaline-2,3-dione (CNQX, 50 μM). Notably, we did not observe an ADP-dependent burst of action potentials at the beginning of the current injection as was observed previously^20^, which could be due to a difference in species, recording temperature, or other experimental parameters. CA3 neurons in βarr2^+/+^ animals exhibited a significant reduction in instantaneous frequency for the first two action potentials elicited after mGluR1 activation (18.1±1.6 Hz pre-DHPG, 12.0±1.2 Hz with DHPG, *n*=17; *P*=0.0003, Fig. 4a). A marked reduction also was observed in parallel experiments using βarr2^−/−^ littermates (16.6±0.8 Hz pre-DHPG, 12.6±1.1 Hz with DHPG, *n*=14; *P*=0.0023, Fig. 4b); the reduction in βarr2^+/+^ and βarr2^−/−^ recordings was not different (−33.6±4.3% for βarr2^+/+^, *n*=17; −24.0±5.6% for βarr2^−/−^, *n*=14; *P*=0.23). These results indicate that pharmacological activation of mGlu1 receptors causes slowing of action potential firing in CA3 neurons through a βarr2-independent mechanism.

We also monitored membrane potential during DHPG application. Consistent with observations in dissociated CA3 neurons^21^, we observed a membrane hyperpolarization in CA3 neurons in slice recordings from βarr2^+/+^ mice (−70.4±0.5 mV pre-DHPG, −77.5±0.9 mV with DHPG, *n*=17, *P*=0.0005, representative trace in Fig. 4d). In 7 of 17 cells, hyperpolarization was followed by a membrane depolarization that exceeded the baseline membrane potential and led to spiking (Fig. 4d); among the cells in which this depolarization was not sufficient to evoke spiking, we observed depolarization above baseline within 5 min of DHPG application (−70.9±0.7 mV pre-DHPG, −65.9±1.0 mV with DHPG, *n*=10, *P*=0.006, representative trace in Fig. 4f). DHPG application also produced the initial hyperpolarizing potential in βarr2^−/−^ recordings (−71.1±0.7 mV pre-DHPG, −78.9±1.7 mV with DHPG, *n*=14, *P*=0.0023, representative trace in Fig. 4d). βarr2^+/+^ and βarr2^−/−^ mice responded to DHPG application with hyperpolarizations of equal magnitude (7.1±1.1 mV in wild type, *n*=17; 7.8±2.0 mV in βarr2^−/−^, *n*=14, *P*=0.62, Fig. 4c). In βarr2^−/−^ CA3 pyramidal neurons, DHPG elicited spiking in 7 of 14 cells (Fig. 4d), but the non-spiking cells exhibited no appreciable depolarization above baseline membrane potential within five min of DHPG application (−70.5±0.3 mV pre-DHPG, −68.3±1.5 mV with DHPG, *n*=7, *P*=0.22, representative trace in Fig. 4f). The peak depolarization in membrane potential from baseline measured 3–5 min after DHPG application was, therefore, different between βarr2^+/+^ and βarr2^−/−^ littermates (6.3±1.2 mV in βarr 2^+/+^, *n*=13; 2.2±1.4 mV in βarr2^−/−^, *n*=7, *P*=0.047, Fig. 4e). Thus, βarr2-dependent signalling pathways are but a subset of those that can modulate intrinsic conductances in CA3 neurons. These experiments also confirmed that mGlu1 receptors are present and functional on the plasma membrane in βarr2^−/−^ mice.

### βarr2 mediates PP-LFS, mGluR-LTD of CA1 synapses

Activation of group I mGluRs induces LTD at hippocampal Schaffer collateral-CA1 (Sch-CA1) synapses. This form of plasticity is induced by a long train of paired stimuli delivered at a low frequency (PP-LFS LTD)^22^,^23^ or pharmacological activation by group I mGluR agonist DHPG (DHPG LTD)^22^,^24^,^25^, and is thought to require MAPK/ERK^26^, JNK^27^ and developmentally regulated rapid protein synthesis^22^,^28^. Synaptic modification by PP-LFS is induced by co-existing NMDA receptor-independent (for example, mGluR-dependent) and NMDA receptor-dependent mechanisms^29^. We inhibited NMDA receptors in order to test the role of β-arrestins in mGluR-dependent PP-LFS LTD in comparison studies using P16-P25 βarr knockout mice.

**Figure 5:**
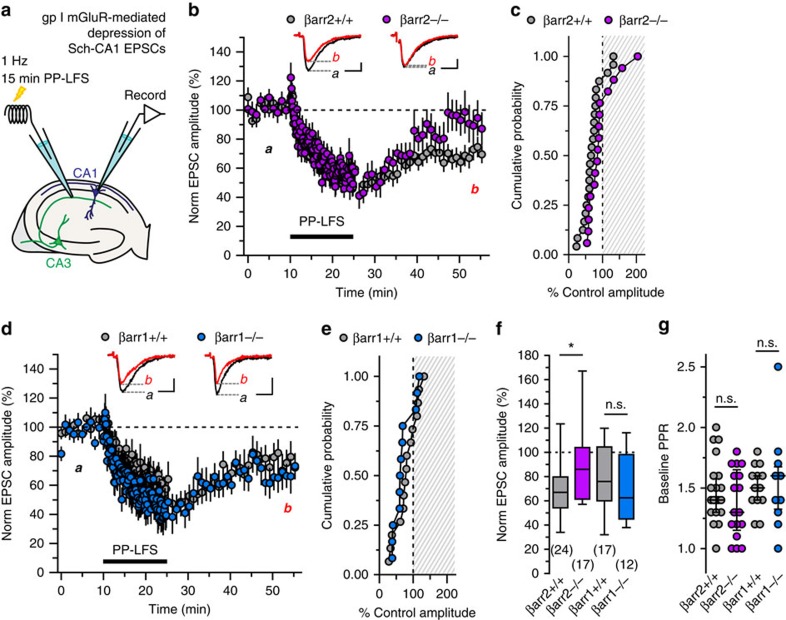
Deficits in CA1 mGluR-LTD in β-arrestin2^−/−^ neurons. (**a**) Whole-cell voltage-clamp recordings in acute hippocampal CA1 slice recordings. Schaffer collaterals in the stratum radiatum received PP-LFS for 15 min, which depresses EPSC amplitudes recorded in CA1 pyramidal neurons. (**b**,**c**) Group I mGluR-mediated depression of Sch-CA1 synaptic transmission is impaired in βarr2^−/−^ mice. A time course plot shows persistent depression of EPSC amplitudes 30 min after PP-LFS in βarr2^+/+^ mice (*n*=24 cells from 17 animals). In contrast, Sch-CA1 EPSCs from βarr2^−/−^ animals recover more than wild types 25–30 min after PP-LFS (*P*=0.03, *n*=17 cells from 10 animals). For illustrative purposes, every third event was plotted during basal stimulation, and every ninth event was plotted during 1 Hz PP-LFS. A cumulative probability histogram illustrates separation of post-PP-LFS amplitudes between βarr2^+/+^ and βarr2^−/−^ recordings. (**d**,**e**) PP-LFS induces equivalent depression of Sch-CA1 EPSCs in βarr1^−/−^ and βarr1^+/+^ (*P*=0.39, *n*=12 cells from seven wild-type animals, *n*=17 cells from 10 βarr1^−/−^ mice), shown in time course and cumulative probability histograms. (**f**) Summary box plot of PP-LFS LTD post-train amplitudes from βarr2 and βarr1 groups. (**g**) Baseline PPRs measured using βarr2- and βarr1-null mice were not different from wild-type littermates (*P*=0.46 for βarr2^+/+^ and βarr2^−/−^, *P*=0.56 for βarr1^+/+^ and βarr1^−/−^). Median and interquartile ranges are provided. Calibration of representative traces: *x* axes, 10 ms; *y* axes, 100 pA. Groups were compared by Mann–Whitney tests. Asterisks denote significance (*P<0.05); n.s., non-significant.

Paired, low-frequency stimulation of Schaffer collateral inputs for 15 min (Fig. 5a) elicits depression of EPSC amplitudes of CA1 neurons in βarr2^+/+^ mice measured 25–30 min after the train (70±6% compared with baseline, *n*=24). In contrast, LTD was absent in recordings from βarr2^−/−^ mice (94±10% compared with baseline, *n*=17, *P*=0.03, Fig. 5b,c,f). PP-LFS induced equivalent levels of LTD in βarr1^+/+^ and βarr1^−/−^ mice (78±8% compared with baseline in βarr1^+/+^, *n*=17; 70±8% in βarr1^−/−^, *n*=12, *P*=0.39, Fig. 5d–f). Baseline paired pulse facilitation was not different when comparing βarr2^−/−^ and βarr1^−/−^ mice with wild-type littermate groups (1.45±0.05 for βarr2^+/+^, *n*=24; 1.38±0.07 for βarr2^−/−^, *n*=17, *P*=0.46; 1.47±0.05 for βarr1^+/+^, *n*=17; 1.56±0.11 for βarr1^−/−^, *n*=12, *P*=0.56, Fig. 5g). These results indicate that mGluR-dependent depression of synaptic transmission in CA1 pyramidal neurons is also dependent on βarr2.

### Sch-CA1 PP-LFS LTD requires mGluR5

**Figure 6:**
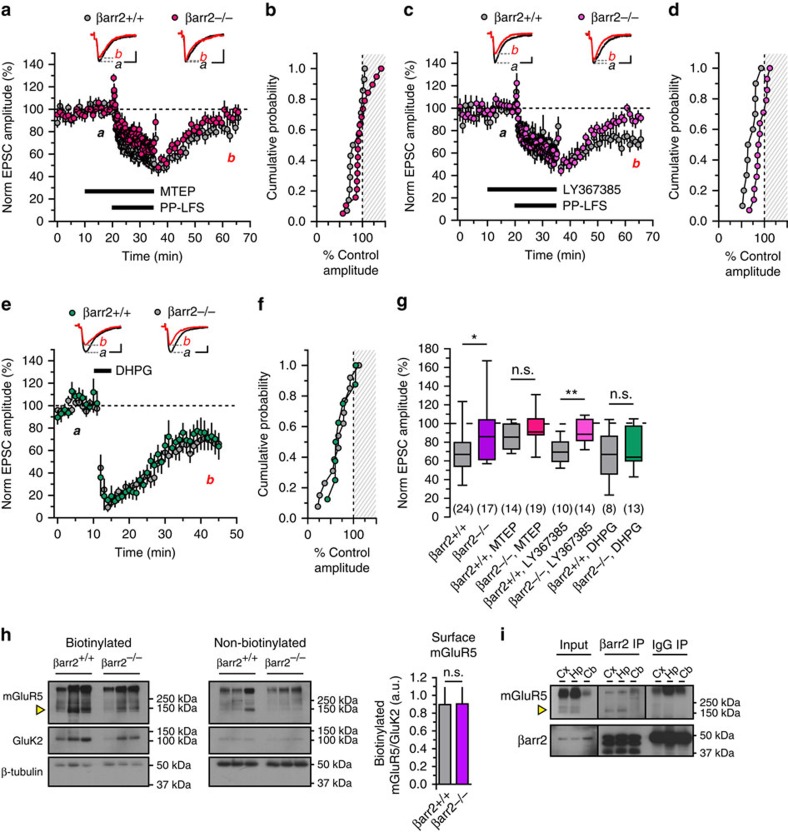
mGluR5 is necessary for LTD induced by synaptic stimulation. (**a**,**b**) Inhibition of mGluR5 by MTEP (1 μM) attenuates prolonged Sch-CA1 synaptic depression in βarr2^+/+^ and βarr2^−/−^ mice. A time course plot shows depression of EPSC amplitudes during PP-LFS in the presence of MTEP, but normalized amplitudes in both βarr2^+/+^ and βarr2^−/−^ animals recover by 30 min after PP-LFS (*P*=0.27, *n*=14 cells from seven βarr2^+/+^ mice, *n*=19 cells from 12 βarr2^−/−^ mice). Every third event was plotted during basal stimulation, and every ninth event was plotted during PP-LFS. A cumulative histogram further summarizes post-train data. (**c**,**d**) Using LY367385 (50 μM), an mGluR1 antagonist, PP-LFS LTD is observed in βarr2^+/+^, but not βarr2^−/−^ mice. A time course plot illustrates depression of EPSCs in βarr2^+/+^ following PP-LFS in the presence of LY367385 and recovery to baseline levels in βarr2^−/−^ animals (*P*=0.003, *n*=10 cells from six βarr2^+/+^ mice, *n*=14 cells from eight βarr2^−/−^ mice); data is summarized in a cumulative histogram. (**e**,**f**) DHPG induces LTD in βarr2^+/+^ and βarr2^−/−^ mice. βarr2^+/+^, and βarr2^−/−^ recordings have similar time courses for depression and post-train amplitude distributions (*P*=0.86, *n*=13 cells from six wild-type mice, *n*=8 cells from four knockout mice). (**g**) A box plot shows recovery of all βarr2^−/−^ conditions from PP-LFS LTD but not DHPG LTD; PP-LFS LTD is abrogated in βarr2^+/+^ recordings only by MTEP. (**h**) Monomeric mGluR5 (yellow arrow) and GluK2 immunoreactivity was detected in both the βarr2^+/+^ and βarr2^−/−^ surface biotinylated fractions by western blotting, and less so in non-biotinylated fractions (*n*=3 pairs of βarr2^+/+^ and βarr2^−/−^ mice). β-tubulin immunoreactivity indicated that intracellular proteins were enriched in the non-biotinylated fraction. Quantitated monomeric mGluR5 optical density normalized against the GluK2 signal is provided in a bar graph illustrating the mean and s.e.m. (**i**) mGluR5 co-immunoprecipitates with β-arrestin2 in the cortex and hippocampus of wild-type mouse brains (*n*=5 blots). Pull-down by an IgG isotype antibody yields little mGlu5 receptor immunoreactivity.β-arrestin2 is strongly detected in the immunoprecipitation and input conditions. Representative traces calibration: *x* axes, 10 ms; *y* axes, 100 pA. Groups were compared by Mann–Whitney tests. Asterisks denote significance (*P<0.05, **P<0.01); n.s., non-significant.

We next conducted similar experiments with the addition of mGluR1 and mGluR5 antagonists in the β-arrestin2 gene-targeted mice to delineate the induction mechanism of PP-LFS-induced LTD at Sch-CA1 synapses. MTEP, a negative allosteric modulator for mGlu5 receptors^30^, was bath applied to acute slice preparations 10 min before and throughout the 15 min PP-LFS train; mGluR5 antagonism by MTEP prevented induction of LTD and resulted in equal recovery of EPSC amplitudes measured 25–30 min post-train in βarr2^+/+^ and βarr2^−/−^ mice (86±4% in wild type, *n*=14; 96±5% in knockout, *n*=19; *P*=0.27, Fig. 6a,b,g). To test for contributions by mGluR1, the competitive mGluR1 antagonist LY367385 (50 μM) was bath applied before and during PP-LFS^31^. EPSCs from neurons in βarr2^−/−^ mice again did not exhibit LTD after PP-LFS (91±3%, *n*=14), whereas those from βarr2^+/+^ animals were depressed (70±4%, *n*=10; *P*=0.003, Fig. 6c,d,g). We, therefore, conclude that PP-LFS induces LTD through activation of mGlu5 receptors, but not mGluR1, and that βarr2 is required to induce this form of plasticity at Sch-CA1 synapses.

### Chemical LTD does not require β-arrestin2

We tested if βarr2 is required for the induction of a chemical form of LTD induced by the group I mGluR agonist DHPG; this form of plasticity is thought to share some signalling elements with that induced by electrical stimulation^22^,^26^. DHPG (100 μM, 5 min) application to acute hippocampal slices from juvenile (P16-P25) mice reduced Sch-CA1 EPSC amplitudes to 67±7% of control in wild-type mice (*n*=13) and 72±8% of control amplitudes in βarr2^−/−^ littermates (*n*=8), which was not statistically different (*P*=0.86, Fig. 6e–g). Thus, βarr2 is not essential to DHPG-induced LTD, in contrast with its role in PP-LFS LTD.

### mGlu5 surface expression is not altered in βarr2^−/−^ mice

Because βarr2 has a well-characterized role in internalization of many GPCRs, we evaluated whether mGluR5 surface localization was affected in βarr2 gene-targeted mice. We incubated hippocampal wild-type and βarr2^−/−^ brain tissue from acute slice preparations in biotin, and enriched for biotinylated surface membrane proteins using streptavidin Sepharose beads. Western blotting of the biotinylated fraction from wild-type and βarr2^−/−^ tissue showed equivalent levels of hippocampal, surface-expressed mGluR5 when normalized to kainate receptor subunit GluK2 (108 kDa; representative immunoblotting of three wild-type and 3 βarr2^−/−^ mice is shown in Fig. 6h, uncropped blots are provided in Supplementary Fig. 3). GluK2 is a surface and membrane-bound protein not known to associate with βarr2 and was, therefore, utilized as a loading control. mGluR5/GluK2 optical density was 0.90±0.19 a.u. for wild type, and 0.90±0.18 a.u. for βarr2^−/−^ (*n*=3 pairs, *P*=1, Fig. 6h). Notably, mGluR5 and GluK2 immunoreactivity was low in the non-biotinylated fraction compared with the biotinylated fraction, but immunoreactivity for β-tubulin (50 kDa), an intracellular cytoskeletal protein, was greater in the non-biotinylated fraction than the biotinylated fraction. These results indicate that streptavidin immunoprecipitation was strongly enriched for biotinylated membrane proteins and that mGluR5 expression is unaltered in βarr2^−/−^ mice. Expression of mGluR5 in total and membrane-enriched fractions also is not grossly affected in βarr2 knockout animals (Supplementary Fig. 4), and thus it is unlikely that the deficit in PP-LFS LTD in βarr2^−/−^ mice can be attributed to loss of surface-expressed mGluR5.

### mGlu5 receptors and β-arrestin2 associate in brain tissue

In the final set of experiments, we evaluated whether β-arrestin2 and mGlu5 receptors biochemically associate in brain tissue. Immunoprecipitation of βarr2 protein from wild-type mice yielded positive immunoreactivity for mGluR5 monomers (150 kDa) in western blots from cortex and hippocampus but not cerebellum. Monomeric mGluR5 protein was not detected following immunoprecipitation by an IgG isotype antibody (*n*=5, representative blot in Fig. 6i, uncropped blot provided in Supplementary Fig. 5). Similarly, mGluR5 was not detected in co-immunoprecipitation studies performed using tissue from mGluR5^−/−^ mice (*n*=3, representative blot in Supplementary Fig. 6). mGlu5 receptor immunoreactivity in lysate preparations from wild-type and mGluR5^−/−^ tissue confirmed the absence of mGluR5, while βarr2 was detected in the immunoprecipitation and input conditions for both genotypes. These data indicate that βarr2 and mGluR5 associate in the hippocampus and cortex.

## Discussion

Here we report that β-arrestin2 scaffolding proteins are required for some forms of mGluR-dependent excitatory synaptic plasticity at two hippocampal synapses. The observation that non-canonical G protein-independent signalling shapes hippocampal synaptic plasticity expands our understanding of the molecular substrates for learning and memory. β-arrestin2 transduces a form of mGluR1-dependent plasticity of mossy fibre-CA3 synaptic currents that requires MAPK/ERK and Src kinases as downstream effectors. In contrast, classical mGluR-independent mossy fibre LTP did not require either β-arrestin isoform. Some cell-intrinsic responses to mGlu1 receptor activation in CA3 pyramidal neurons, specifically reduced initial spike frequency and transient hyperpolarizing potentials, also were not dependent on β-arrestin2, whereas elimination of β-arrestin2 altered rebound depolarization with prolonged DHPG application. In the Sch-CA1 hippocampal pathway, LTD induced by synaptic stimulation is impaired in β-arrestin2 gene targeted mice and prevented by mGluR5 antagonism. These results show that neuronal mGluR stimulation of β-arrestin signalling occurs at multiple sites in the central nervous system and comprise alternate molecular pathways that could be targeted to treat neuropathological conditions involving group I mGluRs.

Why has group I mGluR-β-arrestin2 signalling at central synapses largely escaped detection to date? First, signal transduction by arrestins has gradually gained wider recognition through pioneering studies with a number of family A and family B 7TMRs over the last 15 years, but analogous evidence linking mGluRs and other family C 7TMRs with arrestins is limited: calcium sensing receptor and mGlu7 receptor function are sensitive to β-arrestin knockdown or overexpression^32^,^33^,^34^, whereas GABA_B_ receptors do not appear to associate with or initiate β-arrestin-mediated trafficking or signal transduction^35^. Second, in previous studies G protein-independent signalling has been associated experimentally with mGlu1 receptors rather than mGluR5 (ref. 2), and the latter has received significantly greater scrutiny in part because of its linkage with Fragile X Syndrome^36^. Third, the convergence of G protein- and β-arrestin-mediated pathways on common downstream effectors, especially MAPK and Src kinases, likely obscured contributions from β-arrestin signalling in some experimental models. Fourth, the group I mGluR agonist DHPG appears to preferentially activate G protein signalling while minimally stimulating β-arrestin pathways downstream of mGlu1 receptors^37^,^38^, which could also account for the divergence between DHPG- and synaptic stimulation-induced effects observed in our studies. It will be of interest to determine what roles β-arrestins have in other forms of G protein-independent signalling by mGluRs that have been described in hippocampal pyramidal cells^2^,^3^,^5^, hippocampal interneurons^4^ and cerebellar interneurons^6^.

mGluR1-arrestin studies in non-neuronal heterologous expression systems and primary neuronal cultures provide important insights regarding the relationship between mGluR1 and β-arrestin1 and are useful to interpret the present observations in slices. Recombinant β-arrestin1 translocates to and co-localizes with recombinant mGlu1 receptors with agonist stimulation^39^, and co-localization could reflect association between β-arrestin1 and mGlu1 receptors based on co-immunoprecipitation studies^39^,^40^. β-arrestin1 also contributes to ERK1 and ERK2 phosphorylation stimulated by receptor activation, and sustained pERK, mediated by β-arrestin1, has been linked to promoting cell survival^13^,^14^. Collectively these studies point towards a role for β-arrestin1 in mGlu1 receptor signal transduction that could exist in parallel with the mGlu1-β-arrestin2 signalling manifested as changes in plasticity and intrinsic excitability that we described in the current study. Moreover, splice variations in the carboxy terminus of the mGluRs could confer an additional level of specificity in their interactions with β-arrestin isoforms to differentially impact distinct aspects of cell signalling. While still speculative for the β-arrestins, an analogous degree of specificity occurs for the interacting proteins Homer and tamalin, which specifically associate with the mGluR1a splice variant due to the presence of a binding site not found in other receptor isoforms^41^,^42^.

We propose that PP-LFS induces formation of post-synaptic mGluR1, β-arrestin2, MEK, and Src complexes as a requisite step to strengthen mossy fibre-CA3 synaptic transmission, as β-arrestin2 scaffolding of MAPK/ERK^8^,^43^ and Src^44^ is regulated by other 7TMRs, in addition to other kinases such as JNK^45^ and Akt/GSK3 (ref. 46). Unexpectedly, we found no evidence that c-Raf underlies mGluR1- and β-arrestin2- dependent plasticity, despite the requirement for ERK activation and scaffolding of c-Raf by β-arrestin2 in *in vitro* systems^15^. One micromolar GW5074, which we used to inhibit c-Raf and b-Raf kinases, significantly attenuates pERK upregulation in slices stimulated by phorbol ester application (Supplementary Fig. 7). The data suggest that other GW5074-insensitive MEK activators, such as the mixed-lineage kinases, protein kinase A or PKLD^16^,^17^,^18^, have a predominant role in regulating the ERK module in this context.

How might MAPK/ERK and Src kinase activation produce their effects on mossy fibre EPSCs? In light of a possible presynaptic, mossy fibre terminal locus of expression for intermediate-term potentiation^3^, Src or ERK might target trans-synaptic signalling pathways to modulate vesicle release. For example, Src or MAPK/ERK may target the Eph-ephrin pathway, which has been proposed in classical mfLTP as a critical bidirectional transduction mechanism that bridges post-synaptic induction machinery with a presynaptic locus of expression^47^. MAPK/ERK could also stimulate rapid protein synthesis that ultimately modifies pre- and post-synaptic function. In CA3 neurons, ERK promotes phosphorylation of eukaryotic initiation factor 4E (eIF4E), NMDA receptor 2B (GluN2B) protein levels and transcription of ephrinB2 (ref. 48), thereby achieving indirect modulation of post-synaptic ion channel density and trans-synaptic signalling mechanisms to impact synaptic transmission.

Transduction of mGluR1-dependent synaptic plasticity by β-arrestin2 could also occur through signalling events restricted to the post-synaptic compartment. In CA3 neurons, calcium influx via L-type calcium channels or release from intracellular stores is required for potentiation of mfEPSCs by low-frequency synaptic stimulation^3^, but the precise relationship between post-synaptic calcium mobilization and β-arrestin-mediated signalling mechanisms remains unclear. Calcium influx might contribute to a parallel, G protein- or Homer-mediated pathway to impact plasticity; alternatively, β-arrestin2 association with the calcium effectors calmodulin and CaMKII (ref. 49) could have a role in signal transduction through mechanisms we have not explored in this study. mGluR1-β-arrestin2-Src signalling, perhaps facilitated by an increase in intracellular calcium, could modulate synaptic ionotropic receptor function. For example, transient potentiation of NMDA receptor-mediated currents by pharmacological activation of mGluR1 is GDPβS-resistant and requires Src tyrosine kinases^2^, and PP-LFS at mossy fibre-CA3 synapses produces sustained potentiation of NMDA receptor-mediated currents^3^ while potentiation of the AMPA/kainate mediated component of the mfEPSC requires post-synaptic Src (Fig. 2). mGlu1 receptor regulation of NMDA receptor currents in CA3 pyramidal neurons might, therefore, occur through β-arrestin2 and Src via direct, post-translational modification (for example, phosphorylation) of NMDA receptors to enhance their conductance and thereby influence other forms of mossy fibre-CA3 plasticity^50^. Alternatively, cytoskeletal reorganization of the post-synaptic compartment induced by mGluR1 and β-arrestin2 could occur through regulation of cofilin. mGlu7 receptors, for example, elicit β-arrestin2-dependent ERK phosphorylation and subsequent NMDA receptor current reduction through reduced NMDA receptor surface expression mediated by actin and cofilin^51^. mGluR-β-arrestin2-cofilin regulation of the surface localization of ionotropic receptors might therefore serve as another mechanism of plasticity.

The mGluR1- and βarr2-dependent plasticity we describe here represents a mechanism for synaptic strengthening of mossy fibres that is distinct from previously described mGluR5-dependent forms of post-synaptic NMDA-LTP in several key aspects^10^,^52^. The latter is elicited by a higher-frequency pattern of synaptic stimulation (25 or 50 Hz bursts for ∼1 s), requires activation of NMDA receptors, mGlu5 receptors and A_2A_ adenosine receptors, is G protein-dependent, and leads to a selective and stable potentiation of NMDA receptor EPSCs at mossy fibre synapses^10^,^52^. In contrast, mGlu1 receptor-dependent plasticity is evoked by a longer, low-frequency patterned stimulation in which the precise timing of the paired stimuli appears to be an important parameter, is independent of NMDA receptor activation, requires β-arrestin2, strengthens both AMPA and NMDA receptor components of the mossy fibre EPSC, and is only operative for <1 h following train stimulation^3^. Thus, these forms of plasticity can be viewed as complementary with regards to the duration of their effects on synaptic strength, the molecular mechanisms of induction, and the pattern of mossy fibre stimulation required for potentiation. Within the CA3 pyramidal neuron, there are points of convergence in signalling: both require Src kinase as a necessary component and both lead to enhanced NMDA contributions to mossy fibre EPSCs. Potentiation of mossy fibre NMDA-EPSCs leads to metaplasticity of both mossy fibre^53^ and associational/commissural synaptic inputs^54^, and, therefore, the complementary mGlu receptor-dependent signalling pathways are likely to be key mechanisms for dynamic modulation of excitatory synapses in CA3 pyramidal neurons.

β-arrestin2 also appears to be necessary for synaptic stimulation-induced LTD at Sch-CA1 pyramidal cell synapses. Activation of post-synaptic group I mGluRs by PP-LFS causes LTD that is at least partially supported by presynaptic mechanisms^55^, whereas internalization of AMPA and NMDA receptors occurs post-synaptically^56^. Our data suggest that β-arrestin2 is engaged downstream of mGluR5 activation; the biochemical association that we observed between β-arrestin2 and mGlu5 receptors is consistent with a potential post-synaptic scaffolding role for β-arrestin2. mGluR5 and post-synaptic β-arrestin2 could promote receptor internalization through MAPK/ERK and JNK and by regulation of local synthesis of endocytic machinery such as Arc; MAPK/ERK, JNK1, Arc, and protein synthesis have all been implicated in mGluR LTD^22^,^26^,^27^,^57^. Post-synaptic mGlu5 receptors directly associated with β-arrestin2 could also target trans-synaptic Eph-ephrin pathways^58^ or stimulate endocannabinoid release in the hippocampus by a yet unidentified mechanism^59^. Alternatively, or even concurrently, mGlu5 receptors could stimulate G protein-coupled mechanisms to produce endocannabinoids that then activate CB1 receptors located on excitatory presynaptic terminals^60^. Presynaptic β-arrestin2 could have a critical role in conjunction with CB1 receptors to depress neurotransmitter release, either by mediating receptor internalization^61^ or by formation of an active signalling complex. The multifaceted signal transduction roles that β-arrestin2 could assume in conjunction with mGlu5 receptors, which might involve interplay with endocannabinoid signalling systems and Homer proteins, could have broader implications for neuropsychiatric diseases such as Fragile X syndrome^62^,^63^ and, therefore, merits additional investigation^64^.

Generation of biased ligands that preferentially promote G protein- or arrestin-dependent signalling afford the possibility of unprecedented fine-tuning of signalling downstream of 7TMs. Exploration of ligand bias for mGluR signalling is at a nascent stage, however. In cell culture models of mGluR1 regulation of cell viability, compounds such as DHPG, quisqualate and ACPD (1-amino-1,3-dicarboxycyclopentane) preferentially activate phosphoinositide hydrolysis (indicative of G-protein signalling) and minimally stimulate β-arrestin pathways. In the experiments presented here, DHPG LTD was intact in β-arrestin2 knockout mice, which is consistent with its induction relying on G protein-dependent mechanisms. However, it is also possible that DHPG activation of non-synaptic group I mGluRs led to induction of LTD via a divergence of signalling mechanisms compared with those regulated by PP-LFS. In contrast to DHPG, quisqualate, and ACPD, glutamate and aspartate elicit ‘balanced' G protein and arrestin responses, whereas succinate and glutarate were identified as arrestin-‘biased' agonists^37^,^38^. We also attempted to test if the putative arrestin-biased agonist succinate altered mf-CA3 EPSCs, but 1 mM succinate application had no effect on mfEPSC amplitudes evoked at basal frequency or on the potentiation of EPSCs induced by PP-LFS (Supplementary Fig. 8). Further development appears warranted given our evidence for an important role of β-arrestins in multiple forms of group I mGluR signalling.

Regulation of divergent cellular responses by distinct mGluR signalling modalities suggests that selective targeting of effector pathways with novel drug compounds could be a useful therapeutic strategy. Biased signalling by mGlu1 receptors via G-protein or β-arrestin pathways has been proposed to be regulated by ligands via specific interactions within the ligand binding domain^37^. Whereas selective targeting of mGluR1-β-arrestin1 signalling could be used to regulate cell cycle and potentially treat some forms of cancer^38^,^65^, targeting of specific mGluR1 signalling modes in the nervous system might have some utility in treating conditions such as cerebellar ataxia^66^ and chronic pain^67^. Drugs that interfere selectively with mGluR5-β-arrestin effectors, should those prove to exist, could have potential in therapeutic targeting of Fragile X syndrome^68^. In addition to orthosteric ligands, allosteric modulators may lead to biased signalling properties as well. For example, an allosteric modulator for mGlu5 receptors with antipsychotic properties exhibited stimulus bias, potentially through enhanced coupling to G_q_, was recently described^69^. There are no analogous biased allosteric modulators for mGlu1 receptors available, and consequently additional tool development will be required in order to test the viability of biased allosterism as a therapeutic approach.

These data suggest that unconventional signalling by both group I mGluR subtypes can be found at hippocampal synapses. Further delineation of the synaptic and non-synaptic roles played by β-arrestin1 and β-arrestin2 downstream of group I mGluR activation, enabled by future development of orthosteric ligands or allosteric modulators, could yield new targets for treatment of neurological disease and provide new insights into the molecular and cellular mechanisms of learning and memory.

## Methods

### Hippocampal slice preparation and electrophysiological recordings

For electrophysiological recordings, acute hippocampal slices were prepared from juvenile (P16-P22 for mossy fibre-CA3 patch-clamp recordings, P16-P25 for Sch-CA1 recordings) C57bl/6, βarr1^−/−^, or βarr2^−/−^ mice of either gender, in accordance with Institutional Animal Care and Use Committee-approved protocols. βarr1 and βarr2 gene targeted mouse lines were generously provided by the laboratory of Dr Robert Lefkowitz of Duke University. Genotyping was performed by Transnetyx (Memphis, TN) and heterozygous by heterozygous breeders were used to raise mixed wild-type and knockout litters. In all of the comparative physiological studies, experimenters were blinded to mouse genotype or drug condition until data analysis was completed.

Mice were transcardially perfused with ice-cold sucrose-rich slicing artificial cerebrospinal fluid (aCSF) containing 85 mM NaCl, 2.5 mM KCl, 1.25 mM NaH_2_PO_4_, 25 mM NaHCO_3_, 75 mM sucrose, 25 mM glucose, 10 μM DL-APV, 100 μM kynurenate, 0.5 mM Na L-ascorbate, 0.5 mM CaCl_2_, and 4 mM MgCl_2_, and oxygenated and equilibrated with 95% O_2_/5% CO_2_. Following perfusion, mice were decapitated and brains were sliced in sucrose-aCSF. Horizontal slices (350 μm) preserving the ventral hippocampus were obtained using a Leica VT1200S vibratome (Leica Biosystems, Buffalo Grove, IL). Hippocampal sections were warmed to 30 °C in sucrose-aCSF and returned to room temperature; they were then transferred to oxygenated aCSF containing 125 mM NaCl, 2.4 mM KCl, 1.2 mM NaH_2_PO_4_, 25 mM NaHCO_3_, 25 mM glucose, 2 mM CaCl_2_, and 1 mM MgCl_2_ and maintained under these incubation conditions for at least 1 h before recording. Following incubation, individual slices were transferred to a recording chamber and continuously perfused with oxygenated aCSF at room temperature (25 °C). Hippocampal CA1 and CA3 pyramidal neurons were visually identified using a BX51WI fixed-stage upright microscope (Olympus, Center Valley, PA) and subsequently used for whole-cell patch clamp recordings with a MultiClamp 700A amplifier (Molecular Devices, Sunnyvale, CA).

For voltage clamp recordings, recording electrodes were pulled borosilicate glass with tip resistances of 3–7 MΩ and filled with an internal recording solution containing 95 mM CsF, 25 mM CsCl, 10 mM Cs-HEPES, 10 mM Cs-EGTA, 2 mM NaCl, 2 mM Mg-ATP, 10 mM QX-314, 5 mM TEA-Cl and 5 mM 4-AP, and adjusted to pH 7.3 with CsOH. All cells were held at −70 mV for the duration of voltage-clamp experiments; in addition, series resistance was continuously monitored and compensated 65–70%.

Baseline mossy fibre-CA3 and Sch-CA1 EPSC amplitudes were obtained by delivering paired (40 ms interstimulus interval) electrical stimulation at a basal frequency (0.05 Hz) using a glass monopolar electrode, positioned in the stratum lucidum or the stratum radiatum, respectively (Figs 1a and 5a). In all of the recordings excitatory transmission was isolated using GABA_A_ receptor antagonists bicuculline (10 μM) and picrotoxin (50 μM), and NMDA receptor antagonist D-APV (50 μM). Mossy fibre inputs were verified by short and stable latency, robust paired pulse facilitation during baseline recording (>1.8), and inhibition (>65%) by group II mGluR agonist DCG-IV (1 μm), bath applied at the end of each recording.

Plasticity of Sch-CA1 and mossy fibre-CA3 synapses was induced electrically using previously characterized stimulation protocols. Protocols were executed using pClamp9 software (Molecular Devices) and an A310 Accupulser driving a D53 constant current isolated stimulator (Digitimer, Welwyn Garden City, United Kingdom). Intermediate-term potentiation of mossy fibre EPSCs was induced by low-frequency stimulation consisting of a single train of paired stimuli (40 ms interstimulus interval) delivered at 1 Hz for 2 min, while mossy fibre long-term potentiation was induced by HFS (three 100 Hz trains, 1 s each delivered in 10 s intervals). mGluR-dependent LTD of Sch-CA1 synapses was induced by PP-LFS (1 Hz, 15 min, 50 ms interstimulus interval) or bath application of DHPG (100 μM, 5 min) while in the presence of NMDA receptor antagonist D-APV and GABA_A_R antagonists.

In current clamp recordings, a potassium gluconate internal solution containing 130 mM K-gluconate, 20 mM KCl, 1 mM K-HEPES, 0.2 mM EGTA, 0.3 mM Na-GTP, 4 mM Mg-ATP pH 7.3 was loaded into recording electrodes. After break-in, CA3 neurons were equilibrated with the internal solution for 10 min and maintained in aCSF containing MTEP and CNQX. Membrane potential was held at −70 mV between suprathreshold current injections; the amount of current injected was calibrated to generate between 4–6 action potentials during each pre-DHPG step. Ten trains of action potentials were evoked by current steps provided at 0.05 Hz. Cells were then measured in gap-free for 5 min before DHPG application; during this time the initial current required to hold at −70 mV was not changed. After 5 min of DHPG application, cells were held at −70 mV and 10 trains of action potentials were elicited by the same amount of current injection used before DHPG application.

### Biochemical associations of endogenous proteins

To assay associations between native mGlu1 and mGlu5 receptors with β-arrestin2 proteins, mice were anesthetized with isoflurane and decapitated. Brains were dissected in ice-cold phosphate-buffered saline. Tissue from cortex, hippocampus, and cerebellum were homogenized in lysis buffer containing 25 mM Tris, 150 mM NaCl, 1 mM EDTA, 1 mM phenylmethylsulfonyl fluoride, 1 mM Na_3_VO_4_, 1 mM NaF, 1% Triton X-100, and protease inhibitor cocktail (P2714, Sigma-Aldrich, St Louis, MO) and rotated end over end for 25 min. Solubilized homogenates were then clarified by centrifugation and lysate protein content was measured by Bradford assays using a spectrophotometer (Eppendorf, Hauppauge, NY). One milligram of protein from each sample was then pre-cleared using 40 μl protein A/G beads (20421, Thermo Scientific, Bannockburn, IL) for 1 h, rotating end over end at 4 °C. Pre-clearing beads were discarded and 50 μl of protein A/G bead slurry and rabbit polyclonal anti-βarr2 antibody (0.02 μg μl^−1^; ab31294; Abcam, Cambridge, MA) or rabbit IgG antibody (0.02 μg μl^−1^; DA1E; Cell Signaling Technology, Danvers, MA) were added to the supernatant. Co-immunoprecipitation by βarr2 or rabbit IgG was performed overnight at 4 °C rotating end over end. Proteins immobilized on the antibody-conjugated beads were eluted with a mixture of 2 × Laemmli buffer with β-mercaptoethanol and separated by SDS–polyacrylamide gel electrophoresis in an 8% polyacrylamide gel. Following this, proteins were transferred to a polyvinylidene difluoride membrane, blocked in 5% milk in tris-buffered saline, 0.1% Tween 20 (TBS-T), and immunoblotted using mouse anti-mGluR1 (0.05 μg ml^−1^; 610964; BD Biosciences, San Jose, CA), rabbit anti-mGluR5 (0.2 μg ml^−1^; AB5675; EMD Millipore, Billerica, MA) or rabbit anti-βarr2 (0.125 μg ml^−1^) antibodies. Pulldown of proteins was visualized using HRP-conjugated goat anti-mouse and goat anti-rabbit secondary antibodies (GE Healthcare, Little Chafont, United Kingdom) with enhanced chemiluminescence (ECL) substrate (Pierce ECL Western Blotting Substrate, Thermo Scientific, Bannockburn, IL).

### Surface biotinylation

Acute brain slices were prepared as described for physiology. After 1 h of incubation at room temperature, slices were transferred into 6-well plates containing 0.5 mg ml^−1^ sulfo-NHS-LC-Biotin (21335; Pierce) in aCSF, on ice. Dissected hippocampi were biotinylated for 30 min and were subsequently washed twice with NH_4_Cl (50 mM)-supplemented aCSF and twice with aCSF, then homogenized in 1 ml of homogenization buffer (10 mM Tris, 320 mM sucrose, protease inhibitors, pH=7.4). Samples were clarified by centrifugation at 1,000*g* for 5 min and supernatants were collected for centrifugation at 100,000*g* for 1 h. Pellets were then resuspended in 500 μl lysis buffer containing 20 mM HEPES, 150 mM NaCl, 2 mM EDTA, 1% Triton X-100, 0.1% SDS and protease inhibitors with a pH of 7.4 and sonicated. Samples were rotated end over end for 2 h at 4 °C; following solubilization, samples were centrifuged for 40 min at 100,000*g*. The supernatants were subsequently collected and the protein concentration was measured. Three-hundred micrograms of protein brought to a 300 ml final volume from each mouse was then incubated with 40 μl of washed streptavidin-Sepharose beads (17-5113-01; GE Healthcare) overnight at 4 °C. Streptavidin immunoprecipitation samples were centrifuged at 1,000*g* for 3 min and the supernatant (non-biotinylated fraction) was collected. The Sepharose beads bound to the biotinylated fraction were washed three times with lysis buffer and 150 μl of 2 × Laemmli sample buffer (Bio-Rad) with β-mercaptoethanol was added. The protein samples were agitated and boiled at 95 °C for 5 min and samples were frozen or used immediately for gel electrophoresis. Antibodies raised against mGluR5 (0.2 μg ml^−1^), GluK2 (1:10,000; clone NL9, 04–921; EMD Millipore), and β-tubulin (1:10,000) were used to measure immunoreactivity for the respective proteins by western blotting. Film obtained following western blotting was scanned and optical densities were measured using ImageJ.

### Statistical analysis

Non-parametric Mann–Whitney tests were used to compare mean post-train amplitudes and mean baseline PPRs between genotypes or drug-treated groups; Wilcoxon tests were used to compare baseline versus peak DHPG application responses. Mean±s.e.m. is provided throughout the text. EPSC amplitude time courses are represented as mean±s.e.m., while baseline PPRs are shown with median and inter-quartile ranges. All box and whisker plots are represented as median, interquartile range and 10–90% percentiles.

### Pharmacological compounds

The following compounds were either bath applied with aCSF or used to supplement the recording internal solution during pharmacology experiments: PP2 (20 μM), pp60^cSrc^ 521–533, phosphorylated (200 μM), u0126 (20 μM), GW5074 (1 μM), (*S*)-3,5-DHPG (50–100 μM), MTEP (1 μM), (+)-2-methyl-4-carboxyphenylglycine (LY367385, 50 μM) and CNQX (50 μM). CNQX was purchased from Sigma-Aldrich. DHPG, MTEP and LY367385 were purchased from Abcam; all other pharmacological agents were purchased from Tocris Bioscience (Bristol, UK).

### Data availability

The data that support the findings of this study are available from the authors on reasonable request.

## Additional information

**How to cite this article:** Eng, A. G. *et al*. Transduction of group I mGluR-mediated synaptic plasticity by β-arrestin2 signalling. *Nat. Commun.* **7,** 13571 doi: 10.1038/ncomms13571 (2016).

**Publisher's note**: Springer Nature remains neutral with regard to jurisdictional claims in published maps and institutional affiliations.

## Supplementary Material

Supplementary Information Supplementary Figures 1 - 8

## References

[b1] Niswender C. M. & Conn P. J. Metabotropic glutamate receptors: physiology, pharmacology, and disease. Annu. Rev. Pharmacol. Toxicol. 50, 295–322 (2010). 20055706 10.1146/annurev.pharmtox.011008.145533 PMC2904507

[b2] Benquet P., Gee C. E. & Gerber U. Two distinct signaling pathways upregulate NMDA receptor responses via two distinct metabotropic glutamate receptor subtypes. J. Neurosci. 22, 9679–9686 (2002). 12427823 10.1523/JNEUROSCI.22-22-09679.2002 PMC6757830

[b3] Frausto S. F. . A novel form of low-frequency hippocampal mossy fiber plasticity induced by bimodal mGlu1 receptor signaling. J. Neurosci. 31, 16897–16906 (2011). 22114260 10.1523/JNEUROSCI.1264-11.2011 PMC3241444

[b4] Gee C. E. & Lacaille J. C. Group I metabotropic glutamate receptor actions in oriens/alveus interneurons of rat hippocampal CA1 region. Brain Res. 1000, 92–101 (2004). 15053957 10.1016/j.brainres.2003.11.046

[b5] Heuss C. . G-protein-independent signaling mediated by metabotropic glutamate receptors. Nat. Neurosci. 2, 1070–1077 (1999). 10570483 10.1038/15996

[b6] Kubota H. . mGluR1-mediated excitation of cerebellar GABAergic interneurons requires both G protein-dependent and Src-ERK1/2-dependent signaling pathways. PLoS ONE 9, e106316 (2014). 25181481 10.1371/journal.pone.0106316 PMC4152260

[b7] Luttrell L. M. . Beta-arrestin-dependent formation of beta2 adrenergic receptor-Src protein kinase complexes. Science 283, 655–661 (1999). 9924018 10.1126/science.283.5402.655

[b8] Wei H. . Independent beta-arrestin 2 and G protein-mediated pathways for angiotensin II activation of extracellular signal-regulated kinases 1 and 2. Proc. Natl Acad. Sci. USA. 100, 10782–10787 (2003). 12949261 10.1073/pnas.1834556100 PMC196880

[b9] Daniel H., Levenes C. & Crepel F. Cellular mechanisms of cerebellar LTD. Trends Neurosci. 21, 401–407 (1998). 9735948 10.1016/s0166-2236(98)01304-6

[b10] Kwon H. B. & Castillo P. E. Long-term potentiation selectively expressed by NMDA receptors at hippocampal mossy fiber synapses. Neuron 57, 108–120 (2008). 18184568 10.1016/j.neuron.2007.11.024 PMC2390917

[b11] Gee C. E., Benquet P. & Gerber U. Group I metabotropic glutamate receptors activate a calcium-sensitive transient receptor potential-like conductance in rat hippocampus. J. Physiol. 546, 655–664 (2003). 12562994 10.1113/jphysiol.2002.032961 PMC2342598

[b12] Bain J. . The selectivity of protein kinase inhibitors: a further update. Biochem. J. 408, 297–315 (2007). 17850214 10.1042/BJ20070797 PMC2267365

[b13] Emery A. C. . The protective signaling of metabotropic glutamate receptor 1 Is mediated by sustained, beta-arrestin-1-dependent ERK phosphorylation. J. Biol. Chem. 285, 26041–26048 (2010). 20566651 10.1074/jbc.M110.139899 PMC2924003

[b14] Iacovelli L. . Role of G protein-coupled receptor kinase 4 and beta-arrestin 1 in agonist-stimulated metabotropic glutamate receptor 1 internalization and activation of mitogen-activated protein kinases. J. Biol. Chem. 278, 12433–12442 (2003). 12519791 10.1074/jbc.M203992200

[b15] Luttrell L. M. . Activation and targeting of extracellular signal-regulated kinases by beta-arrestin scaffolds. Proc. Natl Acad. Sci. USA. 98, 2449–2454 (2001). 11226259 10.1073/pnas.041604898 PMC30158

[b16] Marusiak A. A. . Mixed lineage kinases activate MEK independently of RAF to mediate resistance to RAF inhibitors. Nat. Commun. 5, 3901 (2014). 24849047 10.1038/ncomms4901 PMC4046110

[b17] Gomez E., Pritchard C. & Herbert T. P. cAMP-dependent protein kinase and Ca^2+^ influx through L-type voltage-gated calcium channels mediate Raf-independent activation of extracellular regulated kinase in response to glucagon-like peptide-1 in pancreatic beta-cells. J. Biol. Chem. 277, 48146–48151 (2002). 12364324 10.1074/jbc.M209165200

[b18] Sato S., Fujita N. & Tsuruo T. Involvement of 3-phosphoinositide-dependent protein kinase-1 in the MEK/MAPK signal transduction pathway. J. Biol. Chem. 279, 33759–33767 (2004). 15175348 10.1074/jbc.M402055200

[b19] Hsia A. Y. . Evidence against a role for metabotropic glutamate receptors in mossy fiber LTP: the use of mutant mice and pharmacological antagonists. Neuropharmacology 34, 1567–1572 (1995). 8606804 10.1016/0028-3908(95)00115-m

[b20] Brown J. T., Booth C. A. & Randall A. D. Synaptic activation of mGluR1 generates persistent depression of a fast after-depolarizing potential in CA3 pyramidal neurons. Eur. J. Neurosci. 33, 879–889 (2011). 21269340 10.1111/j.1460-9568.2010.07565.x

[b21] Harata N. . Two components of metabotropic glutamate responses in acutely dissociated CA3 pyramidal neurons of the rat. Brain Res. 711, 223–233 (1996). 8680866 10.1016/0006-8993(95)01406-3

[b22] Huber K. M., Kayser M. S. & Bear M. F. Role for rapid dendritic protein synthesis in hippocampal mGluR-dependent long-term depression. Science 288, 1254–1257 (2000). 10818003 10.1126/science.288.5469.1254

[b23] Kemp N. & Bashir Z. I. Induction of LTD in the adult hippocampus by the synaptic activation of AMPA/kainate and metabotropic glutamate receptors. Neuropharmacology 38, 495–504 (1999). 10221753 10.1016/s0028-3908(98)00222-6

[b24] Huber K. M., Roder J. C. & Bear M. F. Chemical induction of mGluR5- and protein synthesis--dependent long-term depression in hippocampal area CA1. J. Neurophysiol. 86, 321–325 (2001). 11431513 10.1152/jn.2001.86.1.321

[b25] Palmer M. J. . The group I mGlu receptor agonist DHPG induces a novel form of LTD in the CA1 region of the hippocampus. Neuropharmacology 36, 1517–1532 (1997). 9517422 10.1016/s0028-3908(97)00181-0

[b26] Gallagher S. M. . Extracellular signal-regulated protein kinase activation is required for metabotropic glutamate receptor-dependent long-term depression in hippocampal area CA1. J. Neurosci. 24, 4859–4864 (2004). 15152046 10.1523/JNEUROSCI.5407-03.2004 PMC6729463

[b27] Li X. M. . JNK1 contributes to metabotropic glutamate receptor-dependent long-term depression and short-term synaptic plasticity in the mice area hippocampal CA1. Eur. J. Neurosci. 25, 391–396 (2007). 17284179 10.1111/j.1460-9568.2006.05300.x

[b28] Nosyreva E. D. & Huber K. M. Developmental switch in synaptic mechanisms of hippocampal metabotropic glutamate receptor-dependent long-term depression. J. Neurosci. 25, 2992–3001 (2005). 15772359 10.1523/JNEUROSCI.3652-04.2005 PMC6725134

[b29] Oliet S. H., Malenka R. C. & Nicoll R. A. Two distinct forms of long-term depression coexist in CA1 hippocampal pyramidal cells. Neuron 18, 969–982 (1997). 9208864 10.1016/s0896-6273(00)80336-0

[b30] Cosford N. D. . 3-[(2-Methyl-1,3-thiazol-4-yl)ethynyl]-pyridine: a potent and highly selective metabotropic glutamate subtype 5 receptor antagonist with anxiolytic activity. J. Med. Chem. 46, 204–206 (2003). 12519057 10.1021/jm025570j

[b31] Clark B. P. . (+)-2-Methyl-4-carboxyphenylglycine (LY367385) selectively antagonises metabotropic glutamate mGluR1 receptors. Bioorg. Med. Chem. Lett. 7, 2777–2780 (1997).

[b32] Bouschet T. . The calcium-sensing receptor changes cell shape via a beta-arrestin-1 ARNO ARF6 ELMO protein network. J. Cell Sci. 120, 2489–2497 (2007). 17623778 10.1242/jcs.03469 PMC3324777

[b33] Iacovelli L. . Selective regulation of recombinantly expressed mGlu7 metabotropic glutamate receptors by G protein-coupled receptor kinases and arrestins. Neuropharmacology 77, 303–312 (2014). 24148810 10.1016/j.neuropharm.2013.10.013

[b34] Pi M. . Beta-arrestin- and G protein receptor kinase-mediated calcium-sensing receptor desensitization. Mol. Endocrinol. 19, 1078–1087 (2005). 15637145 10.1210/me.2004-0450

[b35] Sudo Y. . GABA(B) receptors do not internalize after baclofen treatment, possibly due to a lack of beta-arrestin association: study with a real-time visualizing assay. Synapse 66, 759–769 (2012). 22517292 10.1002/syn.21565

[b36] Dolen G. & Bear M. F. Role for metabotropic glutamate receptor 5 (mGluR5) in the pathogenesis of fragile X syndrome. J. Physiol. 586, 1503–1508 (2008). 18202092 10.1113/jphysiol.2008.150722 PMC2375688

[b37] Emery A. C. . Ligand bias at metabotropic glutamate 1a receptors: molecular determinants that distinguish beta-arrestin-mediated from G protein-mediated signaling. Mol. Pharmacol. 82, 291–301 (2012). 22584219 10.1124/mol.112.078444 PMC3400838

[b38] Hathaway H. A. . Pharmacological characterization of mGlu1 receptors in cerebellar granule cells reveals biased agonism. Neuropharmacology 93, 199–208 (2015). 25700650 10.1016/j.neuropharm.2015.02.007 PMC4387075

[b39] Dale L. B. . Agonist-stimulated and tonic internalization of metabotropic glutamate receptor 1a in human embryonic kidney 293 cells: agonist-stimulated endocytosis is beta-arrestin1 isoform-specific. Mol. Pharmacol. 60, 1243–1253 (2001). 11723231 10.1124/mol.60.6.1243

[b40] Mundell S. J. . Desensitization and internalization of metabotropic glutamate receptor 1a following activation of heterologous Gq/11-coupled receptors. Biochemistry 43, 7541–7551 (2004). 15182196 10.1021/bi0359022

[b41] Fagni L. . Complex interactions between mGluRs, intracellular Ca^2+^ stores and ion channels in neurons. Trends Neurosci. 23, 80–88 (2000). 10652549 10.1016/s0166-2236(99)01492-7

[b42] Hirose M. . Phosphorylation and recruitment of Syk by immunoreceptor tyrosine-based activation motif-based phosphorylation of tamalin. J. Biol. Chem. 279, 32308–32315 (2004). 15173175 10.1074/jbc.M400547200

[b43] Shenoy S. K. . β-Arrestin-dependent, G protein-independent ERK1/2 activation by the β2 adrenergic receptor. J. Biol. Chem. 281, 1261–1273 (2006). 16280323 10.1074/jbc.M506576200

[b44] Luan B. . Deficiency of a beta-arrestin-2 signal complex contributes to insulin resistance. Nature 457, 1146–1149 (2009). 19122674 10.1038/nature07617

[b45] McDonald P. H. . Beta-arrestin 2: a receptor-regulated MAPK scaffold for the activation of JNK3. Science 290, 1574–1577 (2000). 11090355 10.1126/science.290.5496.1574

[b46] Schmid C. L. & Bohn L. M. Serotonin, but not N-methyltryptamines, activates the serotonin 2A receptor via a beta-arrestin2/Src/Akt signaling complex in vivo. J. Neurosci. 30, 13513–13524 (2010). 20926677 10.1523/JNEUROSCI.1665-10.2010 PMC3001293

[b47] Contractor A. . Trans-synaptic Eph receptor-ephrin signaling in hippocampal mossy fiber LTP. Science 296, 1864–1869 (2002). 12052960 10.1126/science.1069081

[b48] Nateri A. S. . ERK activation causes epilepsy by stimulating NMDA receptor activity. EMBO J. 26, 4891–4901 (2007). 17972914 10.1038/sj.emboj.7601911 PMC2099472

[b49] Xiao K. . Functional specialization of beta-arrestin interactions revealed by proteomic analysis. Proc. Natl Acad. Sci. USA 104, 12011–12016 (2007). 17620599 10.1073/pnas.0704849104 PMC1913545

[b50] Ali D. W. & Salter M. W. NMDA receptor regulation by Src kinase signalling in excitatory synaptic transmission and plasticity. Curr. Opin. Neurobiol. 11, 336–342 (2001). 11399432 10.1016/s0959-4388(00)00216-6

[b51] Gu Z. . Regulation of N-methyl-D-aspartic acid (NMDA) receptors by metabotropic glutamate receptor 7. J. Biol. Chem. 287, 10265–10275 (2012). 22287544 10.1074/jbc.M111.325175 PMC3322988

[b52] Rebola N. . Adenosine A2A receptors are essential for long-term potentiation of NMDA-EPSCs at hippocampal mossy fiber synapses. Neuron 57, 121–134 (2008). 18184569 10.1016/j.neuron.2007.11.023

[b53] Rebola N. . NMDA receptor-dependent metaplasticity at hippocampal mossy fiber synapses. Nat. Neurosci. 14, 691–693 (2011). 21532578 10.1038/nn.2809

[b54] Hunt D. L. . Bidirectional NMDA receptor plasticity controls CA3 output and heterosynaptic metaplasticity. Nat. Neurosci. 16, 1049–1059 (2013). 23852115 10.1038/nn.3461 PMC3740388

[b55] Faas G. C. . Modulation of presynaptic calcium transients by metabotropic glutamate receptor activation: a differential role in acute depression of synaptic transmission and long-term depression. J. Neurosci. 22, 6885–6890 (2002). 12177186 10.1523/JNEUROSCI.22-16-06885.2002 PMC6757876

[b56] Snyder E. M. . Internalization of ionotropic glutamate receptors in response to mGluR activation. Nat. Neurosci. 4, 1079–1085 (2001). 11687813 10.1038/nn746

[b57] Waung M. W. . Rapid translation of Arc/Arg3.1 selectively mediates mGluR-dependent LTD through persistent increases in AMPAR endocytosis rate. Neuron 59, 84–97 (2008). 18614031 10.1016/j.neuron.2008.05.014 PMC2580055

[b58] Piccinin S. . Interaction between Ephrins and mGlu5 metabotropic glutamate receptors in the induction of long-term synaptic depression in the hippocampus. J. Neurosci. 30, 2835–2843 (2010). 20181581 10.1523/JNEUROSCI.4834-09.2010 PMC6633947

[b59] Chevaleyre V. & Castillo P. E. Heterosynaptic LTD of hippocampal GABAergic synapses: a novel role of endocannabinoids in regulating excitability. Neuron 38, 461–472 (2003). 12741992 10.1016/s0896-6273(03)00235-6

[b60] Kawamura Y. . The CB1 cannabinoid receptor is the major cannabinoid receptor at excitatory presynaptic sites in the hippocampus and cerebellum. J. Neurosci. 26, 2991–3001 (2006). 16540577 10.1523/JNEUROSCI.4872-05.2006 PMC6673964

[b61] Ahn K. H. . Distinct roles of beta-arrestin 1 and beta-arrestin 2 in ORG27569-induced biased signaling and internalization of the cannabinoid receptor 1 (CB1). J. Biol. Chem. 288, 9790–9800 (2013). 23449980 10.1074/jbc.M112.438804 PMC3617280

[b62] Bear M. F., Huber K. M. & Warren S. T. The mGluR theory of fragile X mental retardation. Trends Neurosci. 27, 370–377 (2004). 15219735 10.1016/j.tins.2004.04.009

[b63] Contractor A., Klyachko V. A. & Portera-Cailliau C. Altered neuronal and circuit excitability in fragile X syndrome. Neuron 87, 699–715 (2015). 26291156 10.1016/j.neuron.2015.06.017 PMC4545495

[b64] Luscher C. & Huber K. M. Group 1 mGluR-dependent synaptic long-term depression: mechanisms and implications for circuitry and disease. Neuron 65, 445–459 (2010). 20188650 10.1016/j.neuron.2010.01.016 PMC2841961

[b65] Gelb T. . Metabotropic glutamate receptor 1 acts as a dependence receptor creating a requirement for glutamate to sustain the viability and growth of human melanomas. Oncogene 34, 2711–2720 (2015). 25065592 10.1038/onc.2014.231 PMC5853109

[b66] Notartomaso S. . Pharmacological enhancement of mGlu1 metabotropic glutamate receptors causes a prolonged symptomatic benefit in a mouse model of spinocerebellar ataxia type 1. Mol. Brain 6, 48 (2013). 24252411 10.1186/1756-6606-6-48 PMC4225515

[b67] Chiechio S. & Nicoletti F. Metabotropic glutamate receptors and the control of chronic pain. Curr. Opin. Pharmacol. 12, 28–34 (2012). 22040745 10.1016/j.coph.2011.10.010

[b68] Dolen G. . Correction of fragile X syndrome in mice. Neuron 56, 955–962 (2007). 18093519 10.1016/j.neuron.2007.12.001 PMC2199268

[b69] Rook J. M. . Biased mGlu5-positive allosteric modulators provide in vivo efficacy without potentiating mGlu5 modulation of NMDAR currents. Neuron 86, 1029–1040 (2015). 25937172 10.1016/j.neuron.2015.03.063 PMC4443790

