# Supplementary material for: Transduction of group I mGluR-mediated synaptic plasticity by β-arrestin2 signalling
Source: Nat Commun. 2016 Nov 25;7:13571. doi: 10.1038/ncomms13571 (PMC5133636; doi:10.1038/ncomms13571)
Supplement: Supplementary Information — Supplementary Figures 1 - 8 [file ncomms13571-s1.pdf]

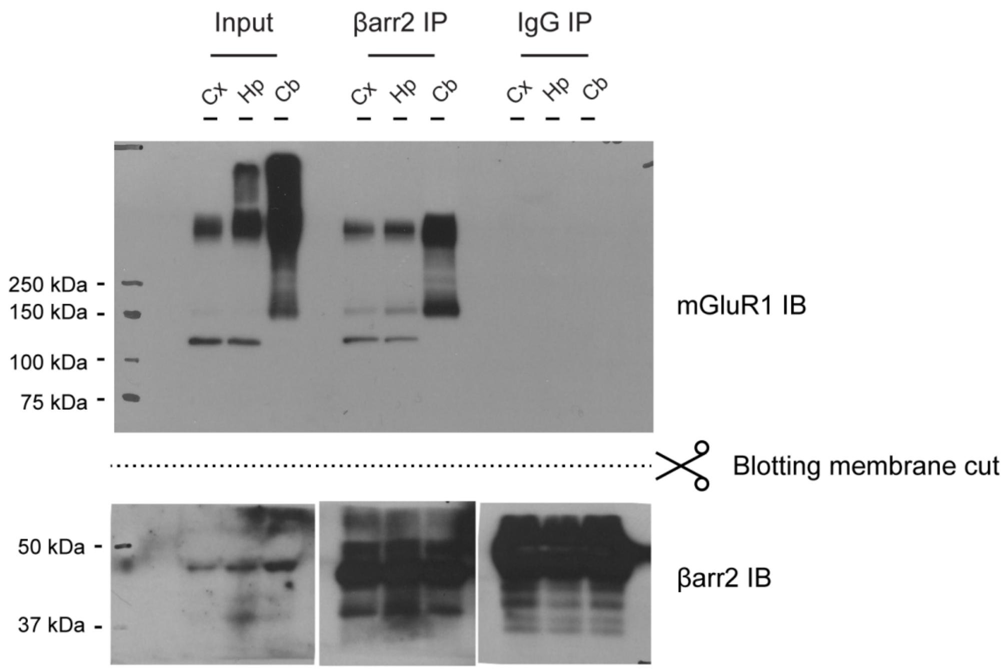

Supplementary Figure 1.

The representative blot from Figure 1i is shown, uncropped, with molecular weight markers.

Figure Legend 1i: mGluR1 coimmunoprecipitates with βarr2 in wild type cortex (Cx), hippocampus (Hp), and cerebellum (Cb), but not with an IgG isotype control antibody (n=3 replicated observations by Western blotting). Positive βarr2 immunoreactivity was observed in immunoprecipitation (IP) and lysate preparations loaded into the same gel. Blotting for βarr2 in the rabbit anti-IgG antibody resulted in strong signal ~50-51 kDa during short exposures, consistent with detection of the rabbit IgG heavy chain by the goat anti-rabbit secondary antibody.

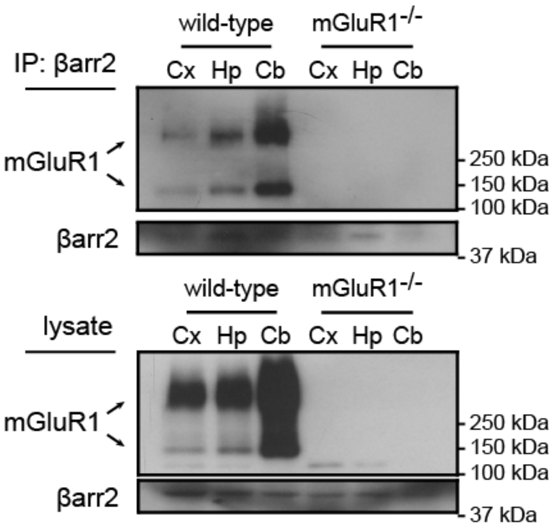

Supplementary Figure 2.

mGluR1 coimmunoprecipitates with βarr2 in wild type cortex (Cx), hippocampus (Hp), and cerebellum (Cb) in wild type, but not mGluR1$^{-/-}$ brain tissue preparations.(n=3 blots). βarr2 expression was verified in the βarr2 immunoprecipitation and input preparations loaded into the same gel.

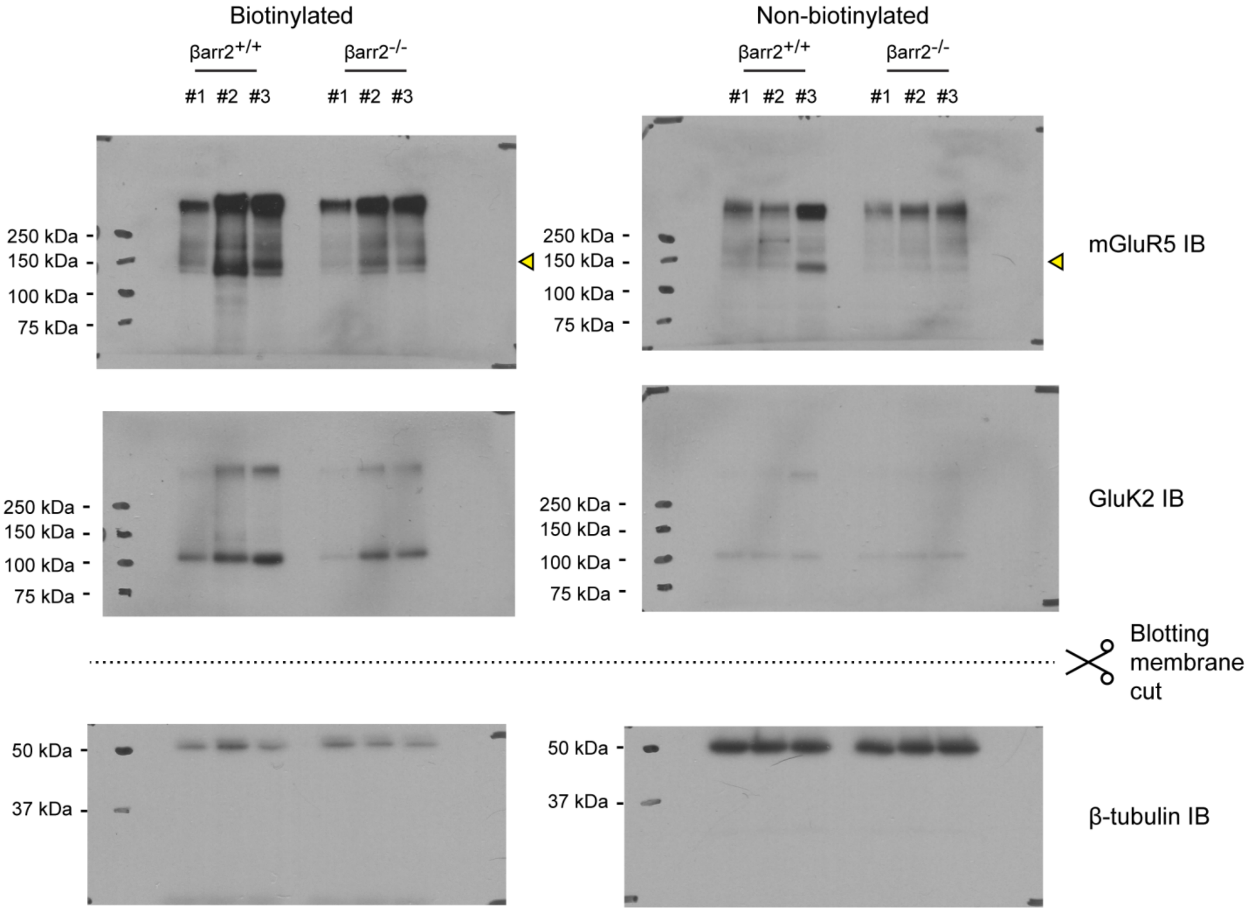

Supplementary Figure 3.

The representative blot from Figure 6h is shown, uncropped, with molecular weight markers.

Figure Legend 6h: Monomeric mGluR5 (yellow arrow) and GluK2 immunoreactivity was detected in both the βarr2$^{+/+}$ and βarr2$^{-/-}$ surface biotinylated fractions by Western blotting, and less so in the non-biotinylated fractions (n=3 pairs of βarr2$^{+/+}$ and βarr2$^{-/-}$ mice). β-tubulin immunoreactivity indicated that intracellular proteins were enriched in the non-biotinylated fraction. Quantitated monomeric mGluR5 optical density normalized against the GluK2 signal is provided in a bar graph illustrating the mean and SEM.

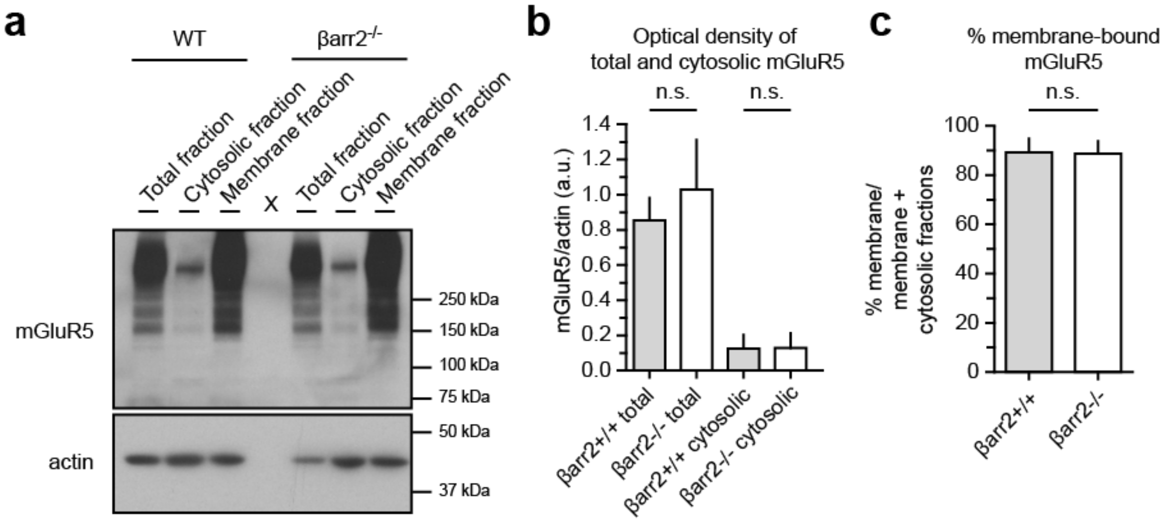

Supplementary Figure 4.

(a) Representative mGluR5 and actin immunoblotting results from wild type and βarr2-null tissue are provided. The blotting membrane containing proteins from the same gel was cut near the 75 kDa marker. The portion of membrane containing proteins 75 kDa or greater was immunoblotted for mGluR5 (~150 kDa, n=2 replicated observations) and robust immunoreactivity was detected in the total and membrane-enriched fractions. In contrast, actin expression was enriched in the cytosolic-enriched fraction relative to the other lysate preparation conditions.

(b-c) Total and cytosolic levels of mGluR5 protein measured as actin-normalized optical density, was unaltered in βarr2-/- brain tissue compared to wild type (0.85 ± 0.13 a.u. for βarr2$^{+/+}$ total fraction normalized to actin, 1.0 ± 0.28 a.u. for βarr2$^{-/-}$ total fraction normalized to actin, and 0.12 ± 0.08 for wild type cytosolic fraction normalized to actin, 0.13 ± 0.08 for βarr2$^{-/-}$ cytosolic fraction normalized to actin n=2). The percentage of membrane-bound mGluR5 was similar in wild type and βarr2$^{-/-}$ brain tissue (89 ± 6% for wild type, 89 ± 5% for βarr2$^{-/-}$, p=0.95, n=2; Mann-Whitney test).

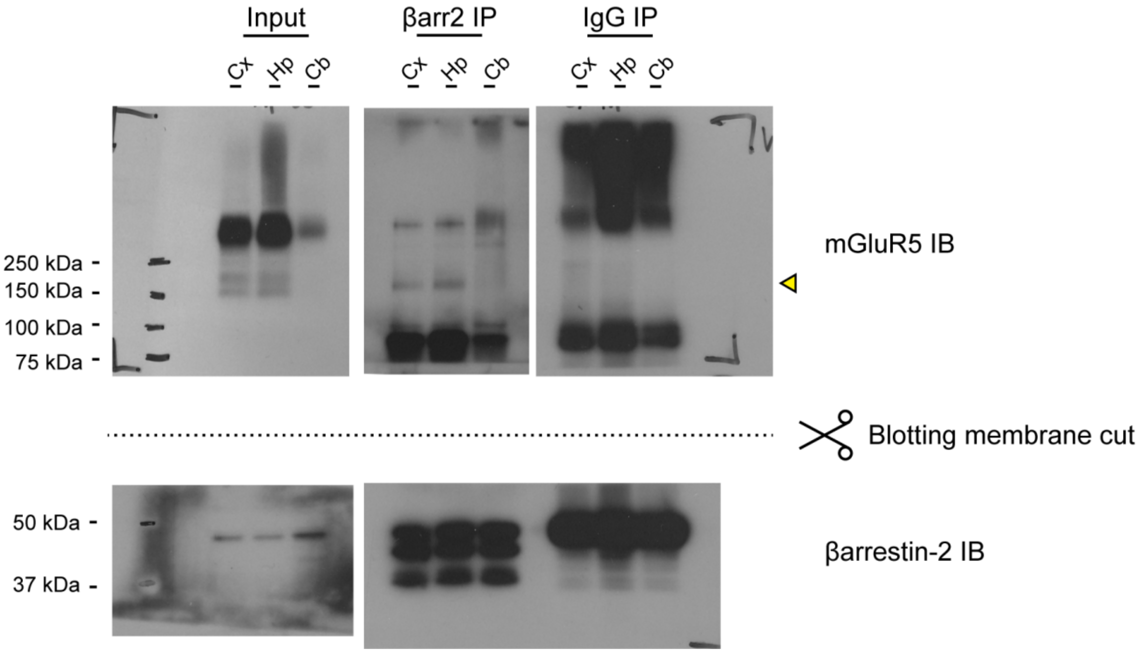

Supplementary Figure 5.

The representative blot from Figure 6i is shown, uncropped, with molecular weight markers.

Figure Legend 6i: mGluR5 receptors co-immunoprecipitate with β-arrestin2 in the cortex and hippocampus of wild type mouse brains (n=5 blots). Pull-down by an IgG isotype antibody yields little mGlu5 receptor immunoreactivity. Robust β -arrestin2 is strongly detected in the immunoprecipitation and input conditions.

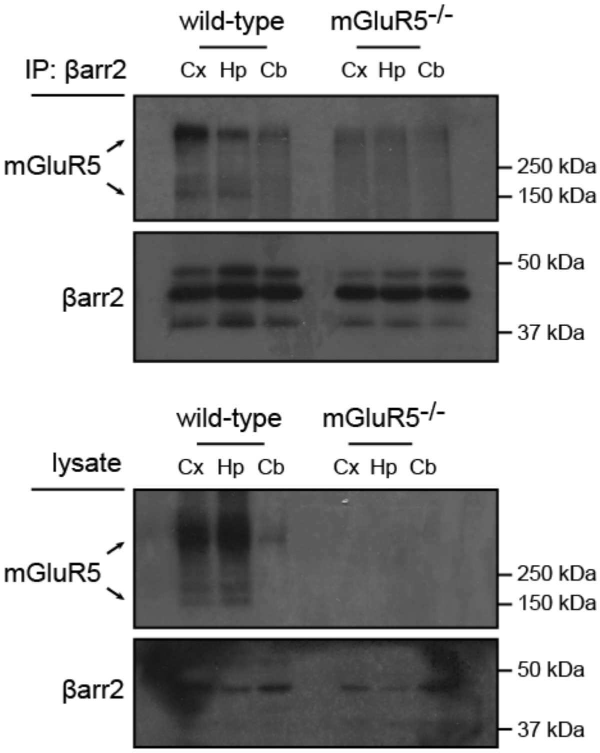

Supplementary Figure 6.

mGlu5 receptors coimmunoprecipitate with βarr2 protein in wild type cortex (Cx), hippocampus (Hp), and cerebellum (Cb), whereas mGluR5 immunoreactivity is not observed in mGluR5$^{-/-}$ mouse brain tissue (n=3 blots). Expression of βarr2 was confirmed in the immunoprecipitation and input protein samples loaded into the same gel.

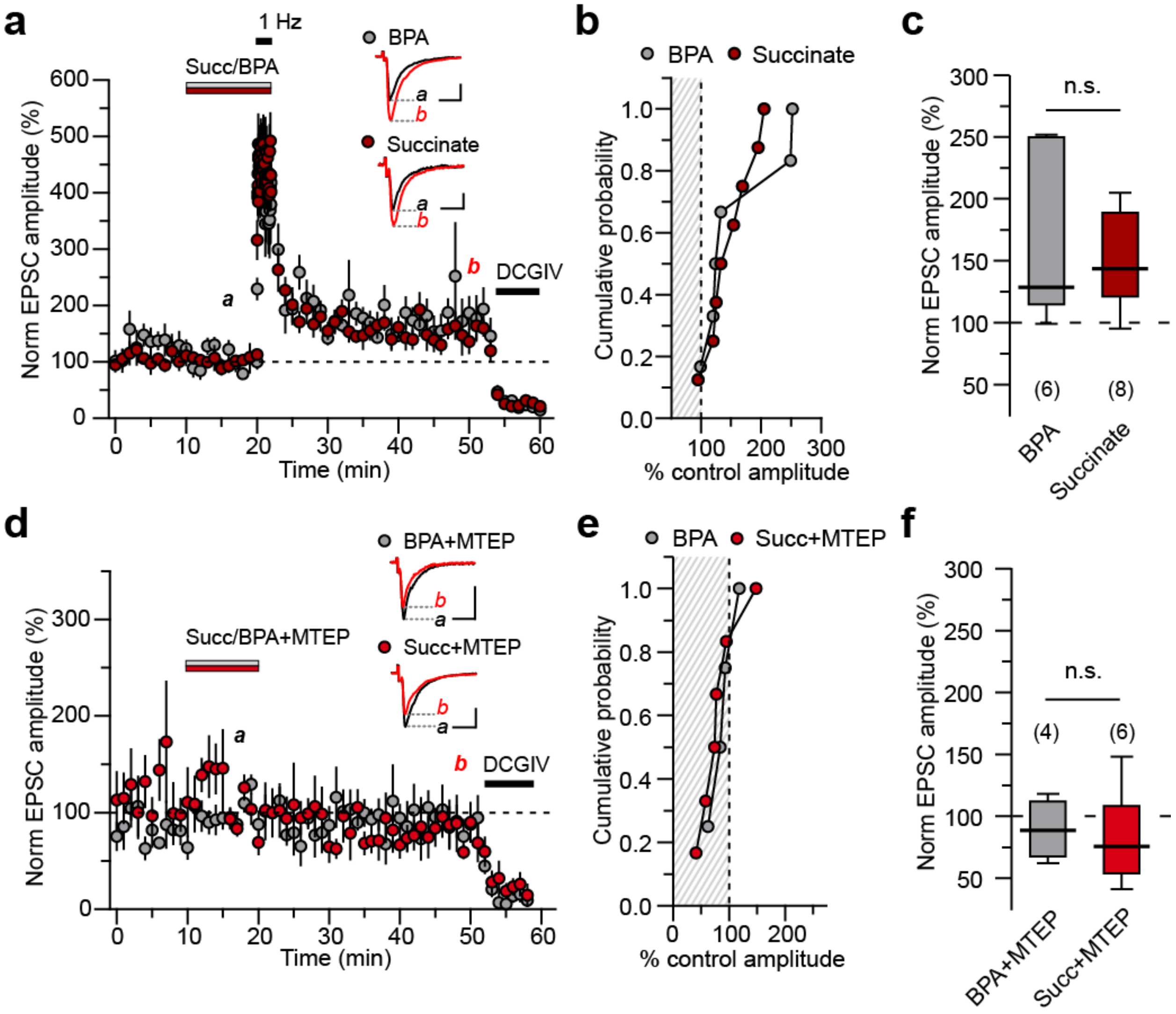

Supplementary Figure 7.

(a-c) Putative arrestin-biased agonist succinate (1 mM) has no effect on plasticity induced by PP-LFS. Post-train amplitudes were 163 ± 28% for vehicle "BPA" treated cells, n=6 cells and 150 ± 14% for succinate treated cells, n=8 cells (p=0.95, Mann-Whitney test). A cumulative probability histogram and box plot summarize the post-train data.

(d-f) Succinate provided in the presence of the mGluR5 antagonist MTEP has no effect on basal mf-CA3 synaptic transmission. Post-application amplitudes were 89 ± 12% for

the vehicle “BPA+MTEP” condition, n=4 cells and 82 ± 15% for the succinate+MTEP condition, n=6 cells (p=0.61, Mann-Whitney test). A cumulative probability histogram and 10-90% box plot summarize the population data for post-train mfEPSC amplitudes. Calibration of representative traces: x-axes, 10 ms; y-axes, 250 pA.

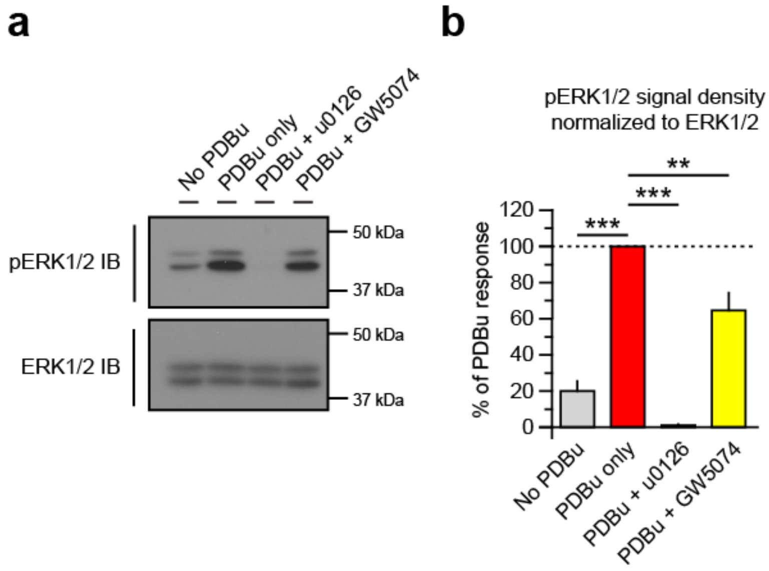

Supplementary Figure 8.

(a) A representative immunoblot of pERK1/2 and ERK1/2 from the same gel and wild type mouse brain are shown. Acute brain slices from the animal were pre-treated for 15 min with u0126 (20 μM, "PDBu + u0126") or GW5074 (1 μM, "PDBu + GW5074") prior to incubation in phorbol 12,13 butyrate for 15 min (PDBu, 1μM). Control "No PDBu" and PDBu-only conditions show basal and maximal pERK signal under these experimental conditions.

(b) Quantitative densitometry analysis of the five independent PDBu experiment replicates illustrates the significant reduction of PDBu-induced pERK upregulation by u0126 and GW5074 compared to treatment with PDBu alone. Phosphorylation of ERK1/2 in the "No PDBu" condition is significantly less than that induced by incubation in PDBu. Quantitated optical densities as a percentage of the PDBu-stimulated pERK immunoreactivity were as follows: 20 ± 5% for PDBu alone, 1.0 ± 0.6% for u0126, and 65 ± 10% for GW5074; the results were compared by One-way ANOVA and post-hoc Tukey multiple comparison tests.

Asterisks denote significant differences between treatment groups (** $p < 0.01$, *** $p < 0.001$).
